# Exploring the Antiproliferative and Modulatory Effects of 1-Methoxyisobrassinin on Ovarian Cancer Cells: Insights into Cell Cycle Regulation, Apoptosis, Autophagy, and Its Interactions with NAC

**DOI:** 10.3390/molecules29081773

**Published:** 2024-04-13

**Authors:** Martina Zigová, Viktória Miškufová, Marianna Budovská, Radka Michalková, Ján Mojžiš

**Affiliations:** 1 Department of Pharmacology, Faculty of Medicine, Pavol Jozef Šafárik University, 040 01 Košice, Slovakia; chripkova.martina@gmail.com (M.Z.); viktoria.miskufova@student.upjs.sk (V.M.); 2 Department of Organic Chemistry, Institute of Chemistry, Faculty of Science, Pavol Jozef Šafárik University, 040 01 Košice, Slovakia; marianna.budovska@upjs.sk

**Keywords:** indole phytoalexins, ovarian cancer, antiproliferative, apoptosis, autophagy, N-acetylcysteine

## Abstract

Ovarian cancer, a highly lethal malignancy among reproductive organ cancers, poses a significant challenge with its high mortality rate, particularly in advanced-stage cases resistant to platinum-based chemotherapy. This study explores the potential therapeutic efficacy of 1-methoxyisobrassinin (MB-591), a derivative of indole phytoalexins found in Cruciferae family plants, on both cisplatin-sensitive (A2780) and cisplatin-resistant ovarian cancer cells (A2780 cis). The findings reveal that MB-591 exhibits an antiproliferative effect on both cell lines, with significantly increased potency against cisplatin-sensitive cells. The substance induces alterations in the distribution of the cell cycle, particularly in the S and G2/M phases, accompanied by changes in key regulatory proteins. Moreover, MB-591 triggers apoptosis in both cell lines, involving caspase-9 cleavage, PARP cleavage induction, and DNA damage, accompanied by the generation of reactive oxygen species (ROS) and mitochondrial dysfunction. Notably, the substance selectively induces autophagy in cisplatin-resistant cells, suggesting potential targeted therapeutic applications. The study further explores the interplay between MB-591 and antioxidant N-acetylcysteine (NAC), in modulating cellular processes. NAC demonstrates a protective effect against MB-591-induced cytotoxicity, affecting cell cycle distribution and apoptosis-related proteins. Additionally, NAC exhibits inhibitory effects on autophagy initiation in cisplatin-resistant cells, suggesting its potential role in overcoming resistance mechanisms.

## 1. Introduction

Ovarian cancer ranks among the prevalent malignant tumors affecting the reproductive organs and has the highest mortality rate among all gynecological malignancies. Approximately 70% of patients are diagnosed with advanced-stage disease, and the majority exhibit resistance to platinum-based chemotherapy, leading to a diminished five-year survival rate [1].

Natural compounds derived from plants have emerged as promising reservoirs of bioactive agents with potential anticancer properties. Among these, phytoalexins, a diverse group of secondary metabolites produced by plants in response to various stressors, have captured the scientific community’s attention due to their multifaceted biological activities [2,3]. A subset of phytoalexins is represented by the indole phytoalexins found in economically significant plants belonging to the Cruciferae family [4]. These compounds are not naturally present in healthy plant tissue but are synthesized de novo in response to pathogen attacks or exposure to physical or chemical stress. Generally, indole phytoalexins serve as integral components of plant defense mechanisms, playing a vital role in safeguarding plants against bacterial and fungal infections [5,6]. Although indole phytoalexins primarily serve to protect plants from biotic and abiotic stress, numerous studies have also demonstrated potential benefits on human health [7,8,9,10]. In addition to other effects, certain studies have reported antiproliferative/anticancer effects of indole phytoalexins either in vitro or in vivo [11,12,13]. 

Within the last two decades, we tested the antiproliferative effects of numerous natural indole phytoalexins and their synthetic derivatives using various cancer cell lines. Our results confirmed the ability of indole phytoalexins to suppress growth and induce cell death in tumor cells. Mechanistically, these effects were associated with the induction of apoptosis, activation of caspases, modulation of the expression of Bcl-2 family proteins, dysregulation of tubulin expression, as well as interference with the activation of signaling pathways, including MAPK or NF-kB. In addition, for some phytoalexins, we demonstrated a correlation between cell death and the induction of oxidative stress [14,15,16,17,18,19,20]. Encouraged by these promising outcomes, we conducted more in-depth research in this field. Over the past five years, we have synthesized and tested a plethora of new derivatives of indole phytoalexins, driven by their remarkable antiproliferative activity [21,22,23,24,25].

**Figure 1:**
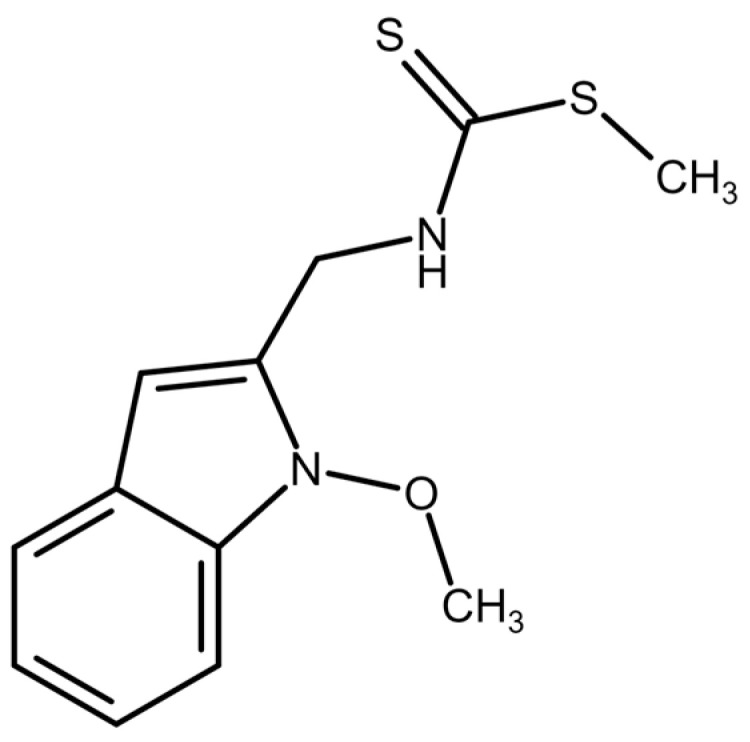
Chemical structure of MB-591 (1-methoxyisobrassinin).

Recently, we documented the synthesis and pronounced antiproliferative effect of the derivative of brassinin, 1-methoxyisobrassinin (Figure 1), on cervical cancer cells [26]. In the presented study, we examined the impact of 1-methoxyisobrassinin (MB-591) on cell proliferation, regulation of the cell cycle, induction of apoptosis, and autophagy in cisplatin-sensitive (A2780) and cisplatin-resistant ovarian cancer cells (A2780 cis). Additionally, we monitored the effect of both N-acetylcysteine (NAC) alone and its combination with the tested indole phytoalexin on these cellular processes. 

## 2. Results

### 2.1. Effect of MB-591 on Metabolic Activity and Viability

**Figure 2:**
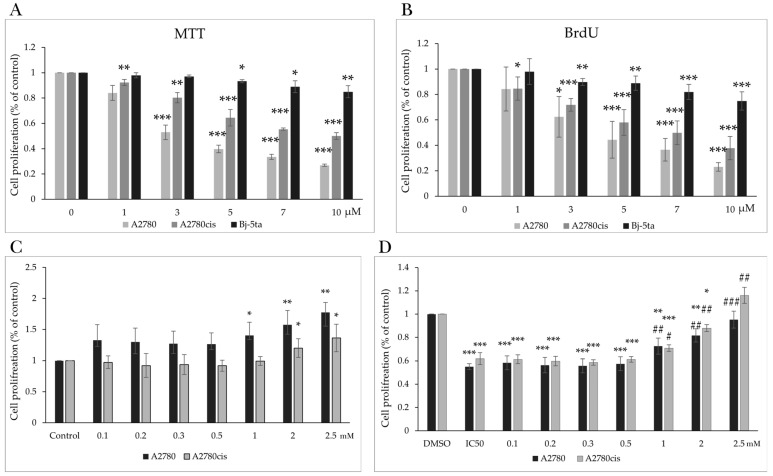
Effect of MB-591 on cell viability and proliferation of A2780, A2780cis and Bj-5ta cell lines. MTT assay (**A**) and BrdU proliferation assay (**B**) after 72 h of MB-591 treatment alone. Effect of NAC on the viability of A2780 and A2780cis cells (**C**) and in combination with the IC50 of MB-591 by MTT assay (**D**). Results show mean ± standard deviation obtained from three independent experiments. Statistical significance: * p < 0.05, ** p < 0.01, *** p < 0.001 vs. control (DMSO) and # *p* < *0.05*, ## *p* < 0.01, ### *p* < 0.001 vs. MB-591.

The effect of MB-591, a studied indole phytoalexin, at varying concentrations on the metabolic activity of select tumor cell lines and a non-tumor cell line was assessed through the implementation of the colorimetric MTT assay. The investigational compound demonstrated marked cytotoxic potential, manifesting a concentration-dependent response in both tumor cell lines. Mitochondrial activity inhibition and diminished cell viability were noted within the concentration range of 3 to 10 µmol/L, exhibiting an IC50 of 3.62 ± 1.19 µmol/L for A2780 cells and 7.00 ± 0.82 µmol/L for A2780cis cells following a 72 h exposure period (Figure 2A).

The selectivity index against the non-tumor cell line Bj-5ta was determined to be 7.57 and 3.91, with corresponding IC50 values of 27.43 ± 2.42 µmol/L. Cisplatin served as the positive control, revealing heightened sensitivity of A2780 cells to cisplatin (IC50 = 1.64 ± 0.35 µmol/L), whereas the IC50 of cisplatin for the cisplatin-resistant A2780cis line escalated to 12.73 ± 2.55 µmol/L. The selectivity index against non-tumor cells was computed as 19.08 for A2780 and 2.45 for A2780cis (Table 1).

In experiments involving NAC, the impact of diverse NAC concentrations (0.1 mmol/L, 0.2 mmol/L, 0.3 mmol/L, 0.5 mmol/L, 1 mmol/L, 2 mmol/L, and 2.5 mmol/L) on cell viability was examined. A noteworthy elevation in viability was discerned from a concentration of 1 mmol/L for A2780 cells and 2 mmol/L for A2780cis cells following a 72 h incubation period with NAC (Figure 2C). A substantial protective effect on cell viability, observed in the concentration range of 1 to 2.5 mmol/L (Figure 2D), suggested that NAC significantly antagonized the cytotoxic effect induced by MB-591 on tumor cells. Subsequently, a non-toxic concentration of NAC, specifically 2.5 mmol/L, was selected for further experimental investigations. 

### 2.2. Effect of MB-591 on Cell Proliferation

Verification of the results of the metabolic MTT assay employed a sensitive colorimetric BrdU Cell Proliferation Assay based on the detection of incorporated 5-bromo-20-deoxyuridine (BrdU) into newly synthesized DNA during the S phase of replicating cells. Results obtained by measuring changes in absorbance in control and treated cells revealed a significant reduction in the proliferation of the A2780 tumor cell line at a concentration of 3 µmol/L, with an IC50 of 5.41 ± 1.19 µmol/L. Additionally, the compound reduced the proliferation of A2780cis cells at a concentration as low as 1 µmol/L, with an IC50 of 7.06 ± 1.09 µmol/L after 72 h of exposure. The IC50 for the non-tumor cell line Bj-5ta was determined to be 18.11 ± 0.93 µmol/L (Figure 2B and Table 1).

Results are presented as mean ± standard deviation of three independent experiments. The selectivity index is calculated based on MTT or BrdU results as a comparison of malignant and non-malignant cell lines.

### 2.3. MB-591 Induces Cell Cycle Arrest

#### 2.3.1. Cell Cycle Analysis

Flow cytometry enables the elucidation of the mechanism of action of the studied substance on cell proliferation and cell cycle progression in affected cells. The tested indole phytoalexin induced a concentration-dependent alteration in the distribution of the cell cycle. Following a 72 h incubation with MB-591, a significant enlargement of the population in the S-phase and a reduction in the number of cells in the G2/M phase of the cell cycle were observed in A2780 cells compared to the unaffected control. In A2780cis cells, a significant redistribution of the population and cell cycle arrest in the S and G2/M phases occurred at IC12.5, dependent on the concentration, with maximal effects at the IC50 (Table 2A,B and Figure S1). These results suggest that the antiproliferative mechanism of the studied substance is associated with alterations in the regulation of the cell cycle in tumor cells.

#### 2.3.2. Analysis of Cell Cycle-Regulating Proteins

**Figure 3:**
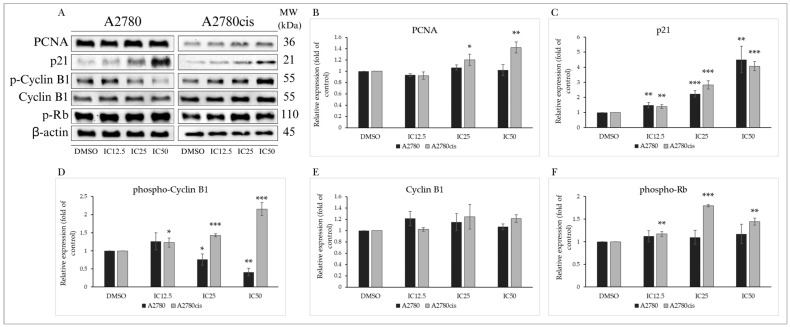
Western blot analysis of cell cycle regulatory proteins after 72 h of MB-591 exposure at IC12.5, IC25 and IC50 concentrations (**A**). Western blot densitometric analysis: relative expression of PCNA (**B**), p21 (**C**), phospho-cyclin B1 and cyclin B1 (**D**,**E**) and phospho-Rb (**F**) in A2780 and A2780cis cells after MB-591 treatment. Representative figure of three independent experiments. Statistical significance: * *p* < 0.05, ** *p* < 0.01, *** *p* < 0.001 vs. cells treated with DMSO (control).

Cell cycle inhibition is closely linked to changes in the expression and activity of proteins associated with cell cycle regulation (Figure 3A). Proliferating cell nuclear antigen (PCNA) is crucial in DNA synthesis and repair, considered essential for cell replication and proliferation. MB-591 did not induce a significant change in the expression of this protein in A2780 cells, but a concentration-dependent (IC25 and IC50) increase in PCNA expression was recorded in A2780cis cells (Figure 3B). Another essential protein regulating cell cycle progression is the promiscuous inhibitor of cyclin-cyclin-dependent kinase complexes, the tumor suppressor p21^Waf1/Cip1^. Through Western blot analysis, a significant increase in p21 expression was observed in both tumor cell lines, with maximal effect at the IC50 concentration after 72 h of exposure to the tested substance (Figure 3C).

Cyclins and their activation through phosphorylation are essential for the activation of cyclin-dependent kinases required for cell cycle progression. Phosphorylation of cyclin B1 at the Ser133 phosphorylation site promotes its nuclear accumulation and initiation of mitosis. In our experiments, MB-591 caused a significant reduction in cyclin B1 phosphorylation without affecting its expression in A2780 cells. Paradoxically, an increase in its phosphorylation was observed in cisplatin-resistant A2780cis cells (Figure 3D,E). The retinoblastoma tumor suppressor protein Rb regulates cell proliferation and the transition through the G1 phase of the cell cycle. Phosphorylation of Rb by cyclin-dependent kinases inhibits its regulatory effect on the transcription factor E2F and contributes to cell cycle progression. The studied indole phytoalexin did not impact the phosphorylation of this protein in the A2780 tumor line after 72 h of incubation. However, a significant concentration-dependent effect was observed in the A2780cis cell line (Figure 3F). The results indicate that MB-591 significantly interfered with cell cycle regulatory mechanisms depending on the concentration of the tested substance and the utilized ovarian tumor cells.

### 2.4. MB-591-Induced Mitochondrial Apoptotic Pathway

#### 2.4.1. Mitochondrial Membrane Potential

**Figure 4:**
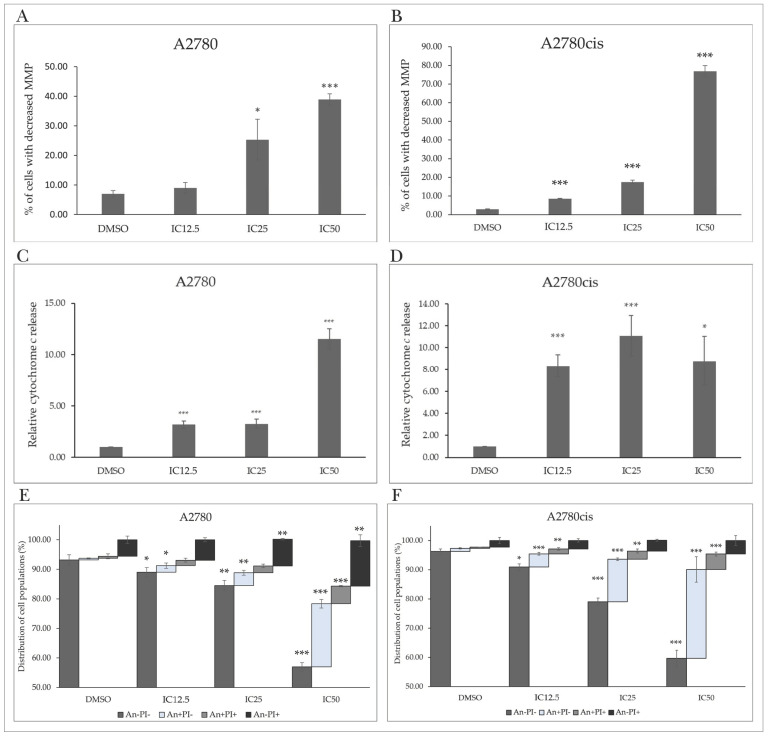
Flow cytometric analysis of apoptosis induced by MB-591 after 72 h in A2780 and A2780cis cells at IC12.5, IC25 and IC50 concentrations. Effect of MB-591 on changes in mitochondrial membrane potential (**A**,**B**), cytochrome c release (**C**,**D**) and externalization of phosphatidylserine (**E**,**F**) using Annexin V/PI staining. Data show mean plus minus SD values from three independent experiments. Statistical significance: * *p* < 0.05, ** *p* < 0.01, *** *p* < 0.001 vs. DMSO (vehicle).

Cell cycle arrest is associated with the induction of regulated cell death through specific proteins. Changes in the ratio of proapoptotic to antiapoptotic proteins facilitate damage and permeabilization of the outer mitochondrial membrane (MOMP). As mitochondria play an essential role in cellular metabolism, survival, and death, their impairment has a lethal effect on the cell. A characteristic indicator of mitochondrial activity is the mitochondrial membrane potential (MMP), the reduction in or loss of which leads to mitochondrial dysfunction and subsequent apoptosis. Exposure of A2780 and A2780cis cells resulted in a significant increase in the number of cells with reduced MMP, with maximal effect at the IC50 after 72 h (Figure 4A,B and Figure S2).

#### 2.4.2. Cytochrome c Release

After MOMP damage, pro-apoptotic factors are released from the intermembrane space into the cytosol, promoting apoptotic stimuli and triggering the death cascade. Our experimental results confirm that the tested indole phytoalexin induced a significant increase in cytosolic cytochrome c levels in cells of both tumor lines at all tested concentrations (Figure 4C,D).

#### 2.4.3. Phosphatidylserine Externalization

Markers of ongoing apoptosis include changes in the fluidity and arrangement of the cell membrane, which remains intact in cells undergoing apoptosis. The anionic phospholipid phosphatidylserine (PS) is localized on the cytosolic side of the lipid bilayer in non-apoptotic cells. Its externalization can be detected using Annexin V (An). Propidium iodide (PI) is a fluorescent membrane-impermeable intercalating dye that accumulates in the nuclei of dead cells. Double-staining with An/PI allows the differentiation of cells into live cells (An-/PI-), apoptotic cells externalizing PS but negative for PI (An+/PI-), doubly positive apoptotic cells (An+/PI+), and necrotic and dead cells (An-/PI+). The population of viable cells after incubation with MB-591 was significantly reduced at each concentration used. The population of An-/PI- A2780 cells at IC50 decreased from 93.17 ± 1.75% to 56.97 ± 1.54%, and the population of An+/PI- increased from 0.55 ± 0.17% to 21.37 ± 1.47%. Similarly, in the cisplatin-resistant A2780cis line, there was a decrease in the An-/PI- population from 96.23 ± 0.86% to 59.73 ± 2.72% at IC50, and the An+/PI- population increased from 1.07 ± 0.23% to 30.33 ± 4.34%. A similar trend was observed in the An+/PI+ cell population exposed to the studied substance (Figure 4E,F and Figure S3).

#### 2.4.4. Changes in Levels of Proteins Associated with the Regulation of Apoptotic Cell Death

**Figure 5:**
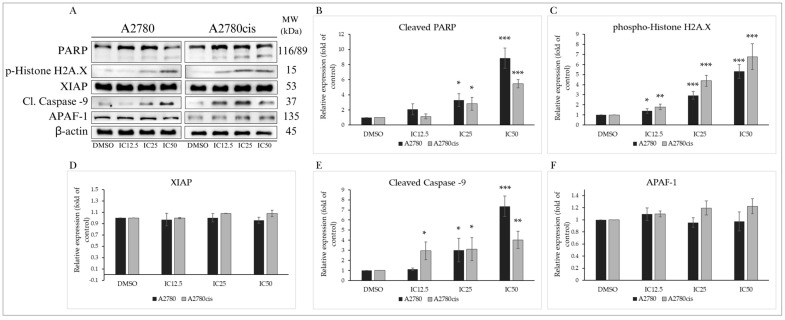
Western blot analysis of proteins associated with apoptosis after 72 h of treatment with MB-591 at IC12.5, IC25 and IC50 concentrations (**A**). Western blot densitometric analysis: relative expression of PARP (**B**), phospho-histone H2A.X (**C**), XIAP (**D**), cleaved caspase -9 (**E**) and APAF-1 (**F**) in A2780 and A2780cis cells after MB-591 treatment. Representative figure of three independent experiments. Statistical significance: * p < 0.05, ** *p* < 0.01, and *** *p* < 0.001 vs. cells treated with DMSO (control).

Apoptotic cell death, besides mitochondrial damage and release of proapoptotic factors into the cytosol, is characterized by changes in protein levels considered members of the caspase cascade, members of the inhibitor of apoptosis (IAP) family, DNA damage markers, and other proteins. One of the essential proteins in the formation of the apoptosome is Apoptotic protease activating factor 1 (APAF-1). Its cytosolic form, in the presence of cytochrome c, forms a complex with caspase-9, which is cleaved, leading to the activation of executioner caspases-3 and -7. We observed an insignificant change in APAF-1 expression, but levels of cleaved caspase-9 were significantly increased with escalating concentration in both A2780 and A2780cis lines (Figure 5A,E,F). Levels of XIAP, a member of the IAP family, were not significantly altered (Figure 5D). Caspase activation leads to the proteolytic cleavage of the DNA repair enzyme poly-(ADP-ribose)-polymerase (PARP). Its inactivation prevents DNA repair and cell survival. Our experimental results demonstrate that the indole phytoalexin MB-591 is capable of increasing levels of cleaved, inactive PARP with increasing concentration after 72 h of exposure (Figure 5A,B). DNA damage is also indicated by a significant increase in phosphorylation of Histone H2A.X, which plays a significant role in DNA repair and response to apoptotic signals. The elevation of phosphorylation at the Ser139 site is the cell’s response to genotoxic stress and double-strand DNA breaks, followed by the activation of additional stress kinases. As depicted in Figure 5A,C, the tested substance significantly increased the phosphorylation of Histone H2A.X in both ovarian tumor cell lines A2780 and A2780cis. The most significant effect was observed at the highest concentration, IC50.

### 2.5. Effect of the Indole Phytoalexin MB-591 on Autophagy Induction

#### 2.5.1. PTEN/Akt/mTOR Signaling Pathway

**Figure 6:**
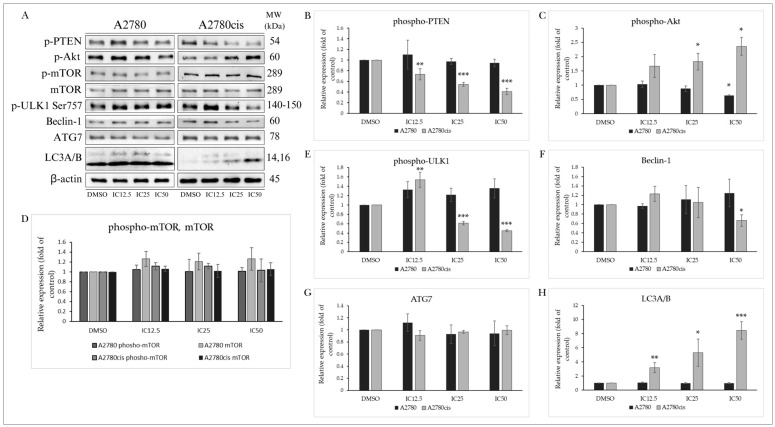
Western blot analysis of autophagy-related proteins after 72 h of MB-591 treatment at IC12.5, IC25 and IC50 concentrations (**A**). Western blot densitometric analysis: relative expression of phospho-PTEN (**B**), phospho-Akt (**C**), phospho-mTOR and mTOR (**D**), phospho-ULK1 (**E**), Beclin-1 (**F**), ATG7 (**G**) and LC3A/B (**H**) in A2780 and A2780cis cells. Representative figure of three independent experiments. Statistical significance: * *p* < 0.05, ** *p* < 0.01, *** *p* < 0.001 vs. cells treated with DMSO.

The protein PTEN (phosphatase and tensin homolog deleted on chromosome ten) is one of the main negative regulators of the PI3K/Akt/mTOR signaling pathway. This tumor suppressor, often mutated in various cancers, is suppressed by phosphorylation, losing its phosphatase activity and tumor suppressor function. In our experiments, we monitored the effect of MB-591 on the phosphorylation status at Ser380, which was not significantly altered in A2780 tumor cells, unlike A2780cis cells, where the level of phosphorylated PTEN was significantly reduced even at the lowest used concentration (IC12.5) (Figure 6A,B). The protein Akt plays a significant role in regulating cellular processes such as proliferation, growth, survival, cell metabolism, angiogenesis, and the cell cycle. Downstream, it can regulate the phosphorylation of its direct substrate mTOR (mammalian target of rapamycin), considered one of the key transcription factors and negative regulators of autophagy. Despite the ability of tested MB-591 to reduce levels of phosphorylated Akt in A2780 cells and increase its levels in A2780cis cells after 72 h of incubation, changes in the expression and phosphorylation of mTOR were not significant (Figure 6A,C,D).

#### 2.5.2. Effect on Autophagy-Associated Proteins

The protein ULK1/Atg1 is one of the key proteins participating in the formation of the ULK1 complex. This process is stimulated by various proteins in response to starvation and ATP deficiency. Phosphorylation at Ser757 by mTOR disrupts the interaction between ULK1 and AMPKα (AMP-activated protein kinase), inhibiting autophagy initiation. Our results show a significant decrease in ULK1 phosphorylation at Ser757 in the cisplatin-resistant ovarian cell line A2780cis, indicating ULK1 complex formation (Figure 6A,E). Beclin-1 is also part of this core complex, recruiting autophagic proteins to the pre-autophagosomal structure known as PAS. As depicted in Figure 6A,F, after 72 h incubation with MB-591, there was a significant decrease in this protein in A2780cis cells. A2780 cells did not show significant changes in Beclin-1 expression, similar to ATG7, whose levels did not change significantly in either cell line after 72 h of exposure (Figure 6A,G). The most significant marker of ongoing autophagy is the protein LC3A/B and its conversion from a cytosolic (LC3A) to its conjugated form (LC3B). The MB-591 compound induced an increase in LC3AB expression in a concentration-dependent manner in cisplatin-resistant cells, while A2780 cells did not show significant changes in the expression of this protein (Figure 6A,H). These results suggest that the studied substance is capable of inducing autophagy in resistant ovarian cancer cells without affecting the autophagic process in non-resistant A2780 cells.

### 2.6. MB-591 Induces Oxidative Stress by ROS Production

**Figure 7:**
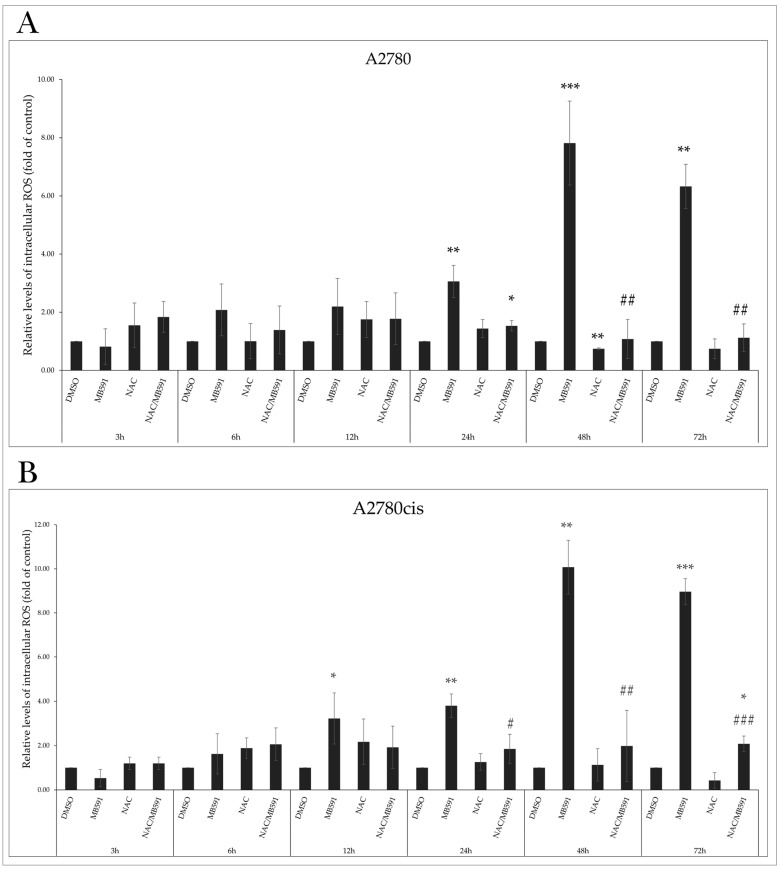
Effect of MB-591, NAC and NAC/MB-591 on ROS production in A2780 (**A**) and A2780cis (**B**) cell lines. Measurement of relative levels of reactive oxygen species levels after 3, 6, 12, 24, 48 and 72 h of incubation. Data ± SD were obtained from three independent measurements. Statistical significance: * *p* < 0.05, ** *p* < 0.01, *** *p* < 0.001 vs. negative control, # *p* < 0.05, ## *p* < 0.01, ### *p* < 0.001 vs. MB-591.

Using flow cytometry, we evaluated the impact of the MB-591 compound, NAC, and their combination on the production of ROS. As it is well known that ROS can cause irreversible damage to DNA, proteins, lipids, and various cell organelles and structures, we monitored changes in intracellular ROS levels alone or in combination with the studied substance MB-591 at different incubation times (3, 6, 12, 24, 48, and 72 h) at the IC50 concentration. In both A2780 and A2780cis cell lines, we observed a significant increase in intracellular ROS levels after 24 and 12 h, which was antagonized in combination with NAC, and compared to cells incubated with MB-591, ROS levels decreased after 24 h (Figure 7A,B).

### 2.7. Effect of NAC on MB-591-Induced Cell Cycle Arrest

Cell cycle analysis after incubation with MB-591, NAC, and their combination showed that in A2780 cells, the studied indole phytoalexin MB-591 induced a significant cell cycle arrest in the S phase (40.05 ± 2.0%) after 6 h compared to control cells (27.95 ± 0.63%). This effect was antagonized by NAC, with a proportion of cells in the S phase at 24.25 ± 3.6%. After 12 h, the cell cycle block shifted to the G2/M phase (39.9 ± 0.42%), persisting until 48 h of incubation with MB-591 (Table 3A, Figure S4A). In the A2780cis line, we observed a significant cell cycle block in the S phase after 3 h of exposure to MB-591 (50.15 ± 4.03%), which also shifted to a G2/M phase block after 12 h with a maximum effect after 48 h of exposure (38.3 ± 1.8%). Incubation with NAC did not affect the cell cycle phase distribution and antagonized the effect of MB-591 in both lines (Table 3B, Figure S4B).

#### Analysis of the Effect of NAC on Cell Cycle-Regulating Proteins

**Figure 8:**
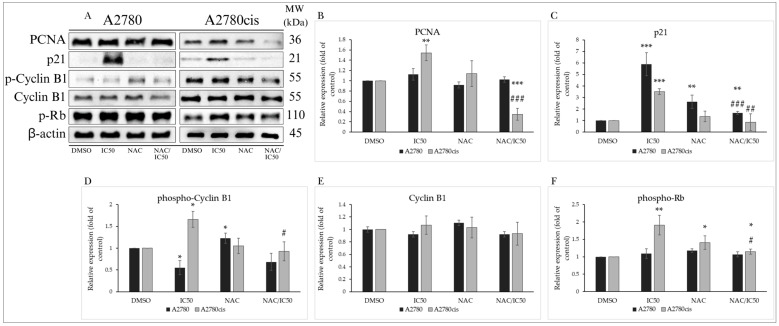
Western blot of cell cycle regulatory proteins after 72 h of treatment with MB-591 at IC50 concentration, NAC and NAC/MB-591 in A2780 and A2780cis analysis (**A**). Western blot densitometric analysis: relative expression of PCNA (**B**), p21 (**C**), phospho-cyclin B1 and cyclin B1 (**D**,**E**) and phospho-Rb (**F**) at A2780 and A2780cis time after MB-591 challenge. Representative figure of three independent experiments. Statistical significance: * *p* < 0.05, ** *p* < 0.01, *** *p* < 0.001 vs. DMSO (control), # *p* < 0.05, ## *p* < 0.01, ### *p* < 0.001 vs. MB-591.

The antagonistic effect of NAC on the action of MB-591 was demonstrated by the analysis of proteins involved in the regulation of the cell cycle. In the A2780cis line, where the studied compound induced an increase in the expression of the key regulatory protein PCNA, NAC at a concentration of 2.5 mmol/L significantly suppressed this effect after 72 h of incubation. Neither MB-591 nor their combination had an impact on PCNA levels in A2780 cancer cells (Figure 8A,B). The increase in levels of the tumor suppressor p21 after exposure to the phytoalexin was significantly suppressed in cells from both cell lines exposed to the NAC/MB-591 combination, reducing them to the levels of control cells (Figure 8A,C). NAC used alone in A2780 cells, unlike the studied substance, increased the phosphorylation of cyclin B1. However, in cells exposed to both NAC and MB-591, it was unable to prevent the MB-591-induced reduction in cyclin phosphorylation. Paradoxically, it inhibited the increase in cyclin B1 phosphorylation in A2780cis cells (Figure 8A,D). Our results also demonstrated the ability of NAC to reduce levels of phosphorylated Rb protein, which were increased after exposure to MB-591 in cisplatin-resistant A2780cis cells. In the A2780 line, we did not observe a significant impact on levels of phosphorylated Rb either alone or in combination (Figure 8A,F). These findings support the results of cell cycle analysis and suggest that the cell cycle block caused by MB-591 is associated with changes in the expression, levels, and phosphorylation of cell cycle-regulating proteins.

### 2.8. NAC Inhibits MB-591-Induced Apoptotic Cell Death

**Figure 9:**
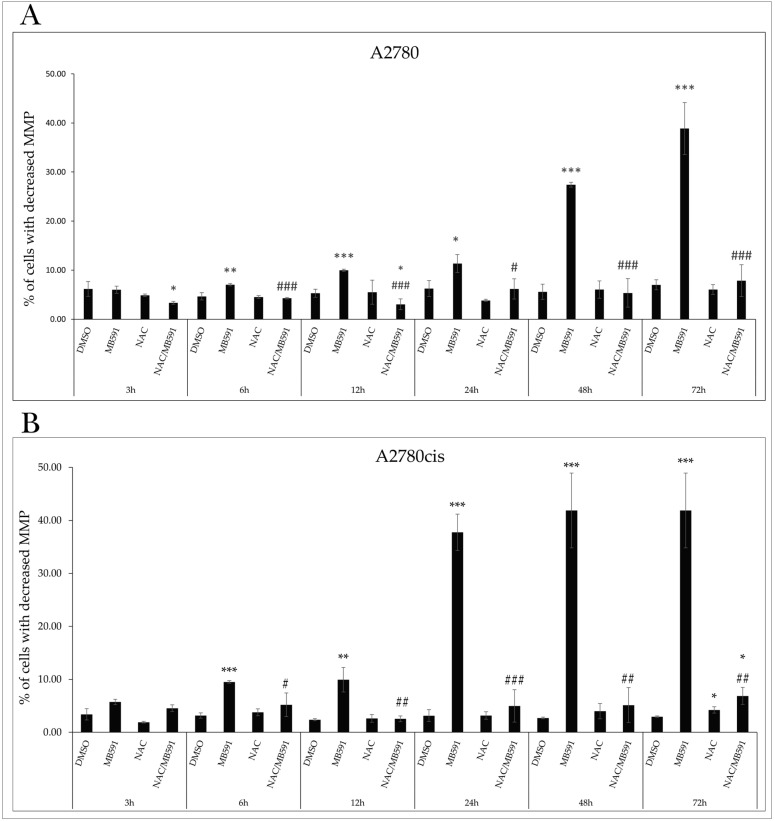
Flow cytometric analysis of the effect of MB-591, NAC/MB-591 on changes in mitochondrial membrane potential and NA after 72 h in A2780 (**A**) and A2780cis (**B**). Data show mean ± SD values of three independent experiments. Statistical significance: * *p* < 0.05, ** *p* < 0.01, *** *p* < 0.001 vs. DMSO (control), # *p* < 0.05, ## *p* < 0.01, ### *p* < 0.001 vs. MB-591.

**Figure 10:**
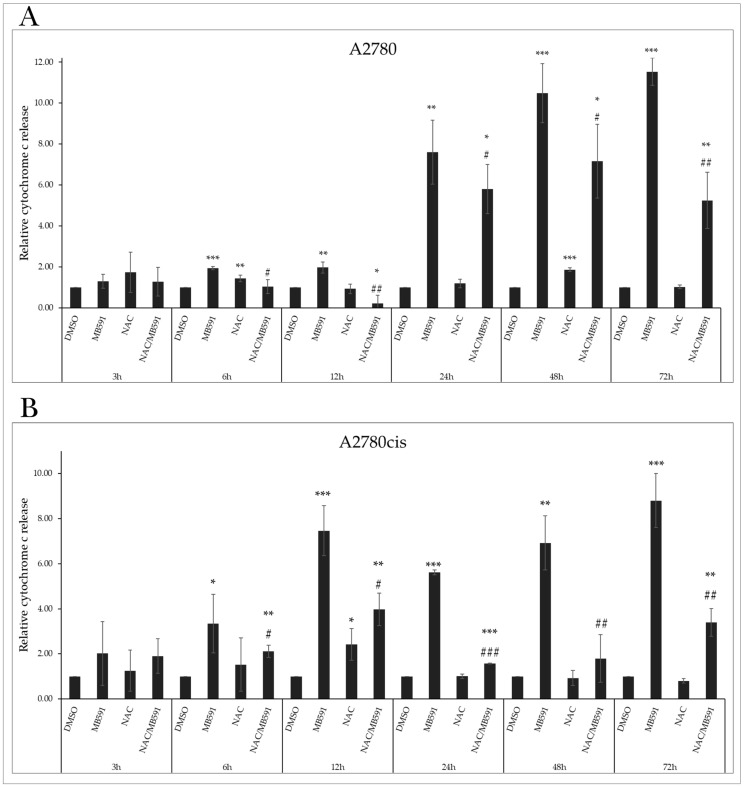
Relative release of cytochrome c after 72 h of treatment with MB-591, NAC and NAC/MB-591 in A2780 (**A**) and A2780cis (**B**). Data show mean ± SD values of three independent experiments. Statistical significance: * *p* < 0.05, ** *p* < 0.01, *** *p* < 0.001 vs. DMSO (control), # *p* < 0.05, ## *p* < 0.01, ### *p* < 0.001 vs. MB-591.

**Figure 11:**
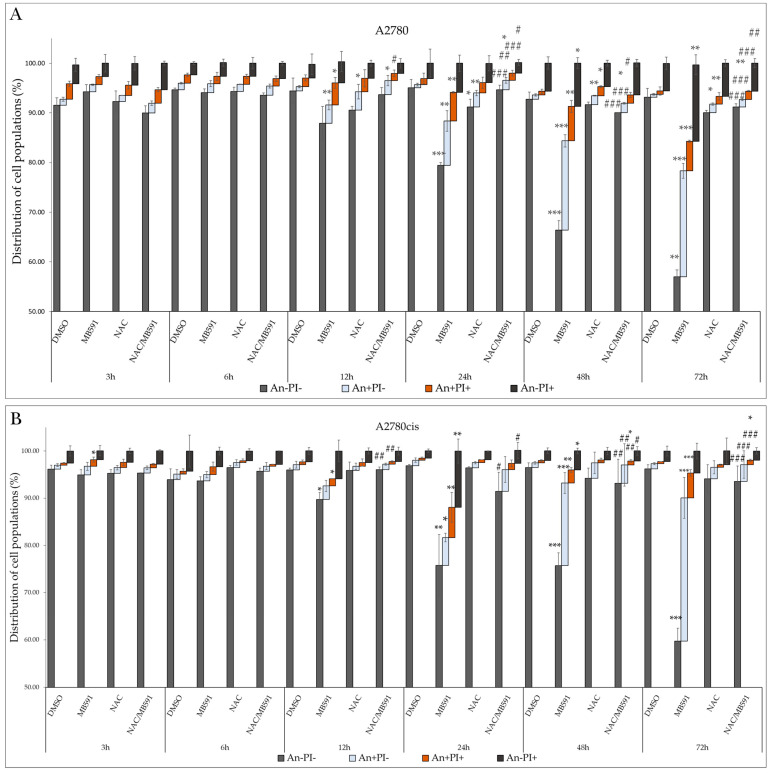
Flow cytometric analysis of phosphatidylserine externalization after 72 h of incubation with MB-591, NAC and NAC/MB-591 in A2780 (**A**) and A2780cis (**B**) assay. Data show mean ± SD values of three independent experiments. Statistical significance: * *p* < 0.05, ** *p* < 0.01, *** *p* < 0.001 vs. DMSO (control); # *p* < 0.05, ## *p* < 0.01, ### *p* < 0.001 vs. MB-591.

As shown in Figure 9A,B (Figure S5A,B), our experiments monitored the impact of the indole phytoalexin MB-591, NAC, and their combinations at various exposure times. As indicated by previous experiments (Figure 4A,B), the studied compound increased the population of cells with reduced MMP. Studying the effect of NAC on this MB-591 effect demonstrated its ability to decrease this population, with a maximum effect after 72 h of incubation, from 38.86 ± 5.2% to 7.8 ± 3.2% in control cells of the A2780 line. This effect was observed from 6 h of exposure. A similar effect of NAC was also observed in the A2780cis line. The reduction in MMP induced by MB-591 is directly related to cytochrome c release, whose presence in the cytosol was significantly increased after 6 h of exposure to the substance at the IC50 concentration for both lines. NAC antagonized this effect at all exposure times, but from 24 h for A2780 cells and 12h for A2780cis cells, the apoptotic effect of MB-591 still prevailed, and cells affected by both NAC and MB-591 simultaneously showed increased relative cytochrome *c* release compared to unaffected cells (Figure 10A,B). Double staining using Annexin V and PI confirmed the aforementioned results, where an increase in the population of cells with externalized Annexin V was recorded after only 12h of exposure to the indole phytoalexin, with a maximum effect after 72 h, 21.37 ± 1.47% for A2780 cells and 30.33 ± 4.34% for the A2780cis line. This effect was significantly antagonized by NAC, which significantly reduced the An+/PI-, An+/PI+, and An-/PI+ subpopulations of cells exposed to the NAC and MB-591 combination (Figure 11A,B, Figure S6A,B).

#### NAC Modulates the Expression of Proteins Involved in Apoptosis

**Figure 12:**
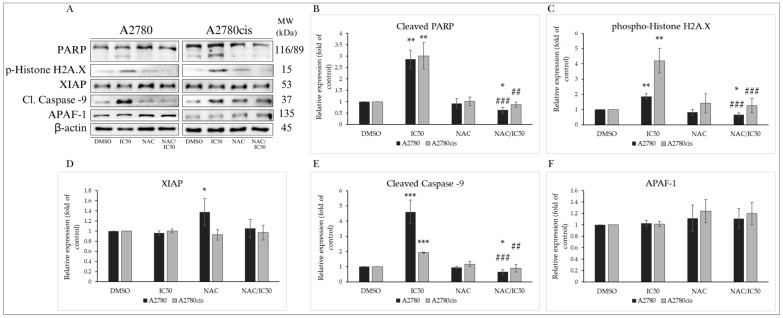
Western blot analysis of proteins associated with apoptosis after 72 h of treatment with MB-591 at IC50 concentration, NAC and NAC/MB-591 in A2780 and A2780cis cells (**A**). Western blot densitometric analysis: relative expression of PARP (**B**), phospho-histone H2A.X (**C**), XIAP (**D**), cleaved caspase-9 (**E**) and APAF-1 (**F**) in A2780 and A2780cis determination. Representative figure of three independent experiments. Statistical significance: * *p* < 0.05, ** *p* < 0.01, *** *p* < 0.001 vs. DMSO (control); ## *p* < 0.01, ### *p* < 0.001 vs. MB-591.

As described in Section 2.4.4, the studied indole phytoalexin induced PARP cleavage, a crucial DNA repair enzyme whose dysfunction leads to irreparable DNA damage and subsequent cell death (Figure 12A,B). NAC was able to reverse this MB-591-induced effect in cells from both lines. Similarly, we observed a significant reduction in the phosphorylation of histone H2A.X, also considered a marker of DNA damage. This effect was monitored through Western blot analysis after 72 h of exposure to MB-591, NAC, and their combination (Figure 12A,C). Despite MB-591 not causing changes in the levels of XIAP and APAF-1 proteins, NAC induced an increase in the expression of these proteins both alone and in cells treated with the NAC/MB-591 combination. However, this effect was observed only in the A2780 cancer cell line (Figure 12A,D,F). Interesting differences were observed in the levels of cleaved caspase 9, essential for initiating the machinery leading to the activation of executioner caspases. In the non-resistant A2780 line, NAC significantly reduced the proapoptotic effect of the studied compound, but in the A2780cis-resistant line, this effect was not as pronounced (Figure 12A,D,F). From these results, it can be inferred that the resistance to cisplatin in A2780cis cells does not affect the antiproliferative effect of the studied indole phytoalexin MB-591 and is equally potent in both ovarian cancer cell lines.

### 2.9. Effect of NAC on MB-591-Induced Autophagy

#### 2.9.1. PTEN/Akt/mTOR Signaling Pathway

**Figure 13:**
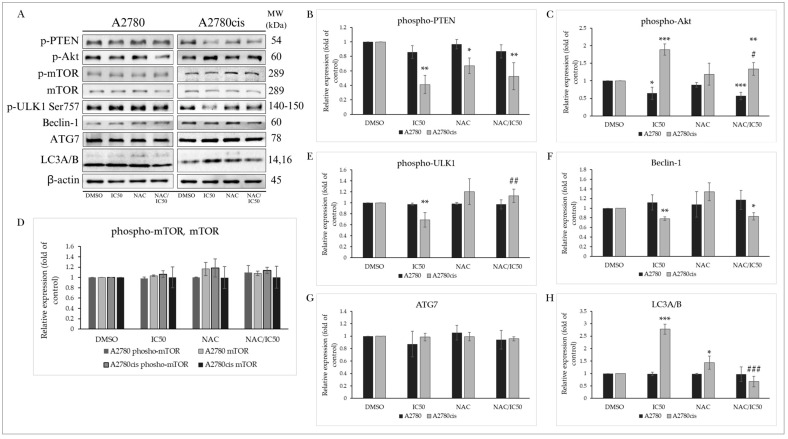
Western blot analysis of autophagy-associated proteins with MB-591, NAC and NAC/MB-591 treatment after 72 h of incubation (**A**). Western blot densitometric analysis: relative expression of phospho-PTEN (**B**), phospho-Akt (**C**), phospho-mTOR and mTOR (**D**), phospho-ULK1 (**E**), Beclin-1 (**F**), ATG7 (**G**) and LC3A/B (**H**) in A2780 and A2780cis. Representative figure of three independent experiments. Statistical significance: * p < 0.05, ** *p* < 0.01, *** *p* < 0.001 vs. DMSO (control); # *p* < 0.05, ## *p* < 0.01, ### *p* < 0.001 vs. MB-591.

As shown by the results of our experiments in Section 2.5.1, the MB-591 compound induced changes in the levels and phosphorylation of proteins involved in the PTEN/Akt/mTOR signaling pathway, mainly in cisplatin-resistant A2780cis cells. This process was characterized by a decrease in PTEN phosphorylation after 72 h of exposure to MB-591, but NAC was able to significantly increase its phosphorylation in cells treated with their combination. Paradoxically, NAC did not have a significant effect on the MB-591-induced increase in phosphorylated Akt levels. In the A2780 cancer cell line, it also did not alter the phosphorylation status of Akt, which was reduced by the phytoalexin (Figure 13A–C).

#### 2.9.2. Effect of NAC on Autophagy-Associated Proteins

The formation of the core complex involves ULK1, known as Atg1, whose phosphorylation was reduced after incubation with the tested substance. This reduction in phosphorylation status, suggesting the initiation of the formation of this complex, was significantly reduced after administration of NAC and also in the NAC/MB-591 combination (Figure 13A,E). This effect indicates NAC-induced inhibition of the autophagic process. Beclin-1, considered a crosstalk between apoptosis and autophagy, is also part of the core complex. NAC could not prevent the MB-591-induced reduction in Beclin-1 levels, and therefore, even in cells exposed to the NAC/MB-591 combination, the Beclin-1 level was reduced after 72 h of exposure (Figure 13A,F). The levels of ATG7 proteins were not changed compared to control cells under all conditions (Figure 13A,G). The most well-known marker of ongoing autophagy is the LC3A/B protein, associated with the formation of autophagic vesicles, which was increased only in the A2780cis line. This effect of the MB-591 compound was significantly reduced in the presence of NAC (Figure 13A,H). Our findings thus suggest that the studied indole phytoalexin MB-591 selectively induces autophagy in cisplatin-resistant ovarian carcinoma cells A2780cis. However, further clarification of the mechanism of this effect is necessary.

## 3. Discussion

The assessment of new agents for treating ovarian cancer is crucial because relapse is common and the recurring disease may show resistance to standard agents, even after the initial response to conventional chemotherapy [27].

Previous research has highlighted the antiproliferative effects of synthetic regioisomeric analogs of isobrassinin and isocyclobrassinin on various cancer cell lines, including A2780. The isocyclobrassinin derivative, characterized by the 1,3-thiazino[5,6-b]indol-4-one structure, displayed the most pronounced antiproliferative activity, achieving 89.6% inhibition at 10 μM, specifically observed in the A2780 cell line. Significantly, this analog demonstrated growth inhibition comparable to that of cisplatin (83.6% at 10 μM) [28,29]. 

In a recent study, we documented the synthesis and pronounced antiproliferative efficacy of 1-methoxyisobrassinin (MB-591) on various cancer cell lines. The highest efficacy was observed on breast cancer cells (MDA-MB-231), with an IC50 of 15.6 µmol/L [26]. So far, however, its effect on ovarian cancer cells has not been studied. 

In our experiments, this indole phytoalexin exhibited pronounced cytotoxicity, demonstrating concentration-dependent responses in A2780 and A2780cis cancer cell lines. The calculated IC50 values highlight a significantly increased potency against the A2780 cell line. Furthermore, the selectivity index against the non-tumor cell line Bj-5ta emphasizes the preferential cytotoxicity of MB-591 towards tumor cells. Cisplatin, employed as a positive control, exhibited increased activity against A2780 cells relative to the cisplatin-resistant A2780cis cell line. 

Oxidative stress plays an important role in cell survival as well as in cell death. Moderate levels of oxidative stress can regulate various cellular processes, plays a role in the immune response or can be involved in hormesis. On the other hand, severe oxidative stress can induce damage of several macromolecules including proteins, nucleic acids, lipids, membranes, and organelles, ultimately resulting in cell death [30,31].

There is abundant evidence that several natural compounds increased production of ROS associated with induction of DNA damage in cancer cells [32,33,34]. DNA damage induces DNA damage response, subsequently affecting the cell cycle, resulting in either DNA repair or apoptosis induction.

The results of our experiments demonstrated that MB-591 induced DNA damage in both cell lines, as evidenced by increased histone H2A.X phosphorylation and PARP cleavage. Additionally, we observed increased production of ROS in both sensitive and resistant tumor cells. Our hypothesis that increased ROS production can be associated with DNA damage is supported by the finding that the antioxidant NAC significantly suppressed not only ROS formation but also markers of DNA damage.

It has been found that numerous natural or synthetic compounds with anticancer properties cause dysregulation of the cell cycle [35,36,37]. Hence, the ability of MB-591 to affect the cell cycle of A2780 and A2780cis cells was evaluated. Flow cytometry analysis revealed that MB-591 induces concentration-dependent alterations in the distribution of the cell cycle. In A2780 cells, it causes cell accumulation at the S phase of the cell cycle after a 72 h incubation at the IC25 concentration. On the other hand, accumulation of A2780cis cells at both the S and G2/M phases of the cell cycle was observed, however, only in cells treated with a higher concentration of MB-591 (i.e., IC50). These findings suggest that the antiproliferative mechanism of MB-591 is closely linked to its impact on cell cycle regulation in tumor cells. This is consistent with previous research demonstrating the ability of indole phytoalexins to modulate cell cycle progression and inhibit cell proliferation in various cancer cell lines. Indole phytoalexins have been shown to exert antiproliferative effects by inducing cell cycle arrest at different phases, including the G1, S, and G2/M phases, depending on the specific compound and cell type studied [12,14,17,18,19].

Cell cycle arrest is closely linked to changes in the expression and activity of proteins associated with cell cycle regulation. We found that MB-591 induces concentration-dependent changes in the expression of several cell cycle regulatory proteins. PCNA plays a multifaceted role in maintaining genomic stability, regulating cell cycle progression, and ensuring the fidelity of DNA replication and repair processes [38]. Our results showed that PCNA shows a concentration-dependent increase in A2780cis cells, with maximal effects at the IC50 concentration. Among others, the increased expression of PCNA has been reported in response to oxidative stress-induced DNA damage [39]. Next, our results also showed that the antioxidant NAC reduced PCNA expression. These findings indicate that increased expression of PCNA may be associated with oxidative DNA damage and the cells’ efforts to repair the damaged DNA. 

Furthermore, it has been reported that increased ROS levels can induce the expression of p21 [40]. This protein also plays a crucial roles in the cellular response to DNA damage via cell cycle arrest and DNA repair [41]. Our results showed a significant increase in p21^Waf1/Cip1^ expression in both cell lines. Recently, an ROS level-dependent function of p21 has been reported. At low levels of cellular ROS, p21 is known to stimulate a cytoprotective response dependent on NRF2. On the other hand, at elevated ROS levels, p21 inhibited the NRF2 activity, ultimately leading to cellular apoptosis [42]. Our results are supported by a recently published study by Acquaviva et al. [43], who found that protocatechuic acid induced apoptosis in colon cancer cells. This effect was associated with the induction of oxidative stress and increased expression of p21.

Additionally, our results showed significantly increased phosphorylation of the RB protein in the resistant ovarian cancer cells. The Rb protein carries out various biological functions such as tumor suppression and cell cycle regulation. In its hypophosphorylated state, Rb binds and inhibits E2F transcription factors, thereby repressing the expression of genes involved in DNA replication and cell cycle progression. Phosphorylation of RB protein by cyclin-dependent kinases releases its inhibition on E2F, allowing for the transcription of genes necessary for cell cycle progression and DNA synthesis [44]. However, under some conditions, increased phosphorylation of RB protein can also be associated with apoptosis induction [45]. In particular, as reported by Ianari et al. (2009) [46], under DNA damage, phosphorylated RB protein participated in the formation of the RB-E2F1 complex, resulting in apoptosis induction.

Cyclins play a crucial role in cell cycle regulation by forming complexes with cyclin-dependent kinases (CDKs), thereby regulating their activity at specific stages of the cell cycle [47]. Cyclin B1 forms a complex with cyclin-dependent kinase 1 (CDK1) to regulate entry into mitosis. Moreover, its phosphorylation is a critical event in regulating cell cycle progression and mitotic entry [48]. In our study, cyclin B1 phosphorylation differed between sensitive and resistant cancer cells. In sensitive MB-591-treated A2780 cells we recorded decreased phosphorylation of cyclin B1. This event reduces the activity of cyclin B1-CDK1 complexes, leading to the inhibition of mitotic entry and induction of the G2/M block. On the other hand, upregulation of phosphorylated cyclin B1 has been observed in resistant A2780cis cells. Although this result is surprising, such differences in cyclin B1 phosphorylation depending on the type of cancer cells have also been documented by other authors [49,50]. These findings underscore the intricate nature of MB-591’s influence on cell cycle regulatory mechanisms, suggesting that the observed effects depend on the substance concentration and the unique characteristics of the cisplatin-resistant cell line.

Poly ADP-ribose polymerase (PARP) is a protein involved in DNA repair mechanisms. When DNA damage occurs, PARP is activated and binds to the site of damage, where it catalyzes the addition of ADP-ribose polymers to itself and other target proteins, facilitating DNA repair [51]. However, in cases of severe DNA damage, excessive activation of PARP can lead to the depletion of cellular NAD+ and ATP, caspase activation, cleavage of PARP and apoptosis. Cleavage of PARP by caspases serves as a marker of apoptosis and is considered an irreversible step in the apoptotic process [52]. 

Moreover, the decrease in MMP is a crucial event in the apoptotic process and is often associated with the activation of intrinsic apoptotic pathways. The loss of MMP facilitates the release of cytochrome *c* from the mitochondrial intermembrane space into the cytosol with subsequent activation of caspase-9 [53]. Our study showed dose-dependent decrease in MMP in MB-591-treated cells. Furthermore, the release of cytochrome *c* as well as cleaved PARP, were significantly elevated in cells exposed to MB-591. In addition, markers of apoptosis, such as phosphatidylserine externalization, revealed a notable reduction in viable cells and an increase in apoptotic and necrotic populations following exposure to MB-591. These results are in line with published studies where several other natural indole phytoalexins or their synthetic derivatives induced apoptosis in various cancer cells by downregulating pro-PARP, activating different caspases, decreasing MMP, and increasing the concentration of ROS [12,17,18,54,55,56].

Autophagy, a crucial cellular process responsible for maintaining homeostasis by eliminating dysfunctional organelles, clearing protein aggregates, and supplying nutrients during stress or nutrient scarcity, is observed to be reduced in cancer cells compared to normal cells and premalignant lesions [57,58,59]. Various molecular pathways, including the autophagy-related gene family, Beclin-1, MAPK, and the PI3K-AKT-mTOR pathways, regulate autophagy [60]. Evidence suggests that targeting autophagy with natural products could be a potential therapeutic approach for cancer [61,62]. However, autophagy induced by chemotherapy or radiotherapy may impede apoptosis, leading to unfavorable conditions post anti-tumor therapy [63]. Detailed mechanisms of different anticancer drug treatments involving the interplay of autophagy and apoptosis remain poorly understood [64].

In our study, we examined the PTEN protein, a primary negative regulator of the PI3K/Akt/mTOR signaling pathway, in relation to the impact of MB-591 on ovarian carcinoma cells. In A2780 cells, its phosphorylation status at Ser380 remained largely unaffected by MB-591. In contrast, A2780cis cells displayed a significant reduction in phosphorylated PTEN even at the lowest concentration (IC12.5 µmol/L). We documented a similar decrease in PTEN phosphorylation and autophagy induction recently in chalcone-treated breast cancer cells [65].

Surprisingly, in cells treated with MB, there were no significant changes observed in the phosphorylation of mTOR, which is the main negative regulator of autophagy. On the other hand, autophagy can be modulated also in an mTOR-independent manner [66]. Furthermore, we monitored the effect of MB-591 on autophagy-associated proteins, specifically ULK1/Atg1, Beclin-1, ATG7, and the key marker LC3A/B. ULK1 phosphorylation at Ser757, a process inhibited by mTOR, is significantly decreased in A2780cis cells, indicating the formation of the ULK1 complex and initiation of autophagy. Beclin-1, an essential component of the core autophagic complex, experiences a significant decrease in expression in A2780cis cells after 72 h of MB-591 exposure. Furthermore, significantly, a dose-dependent increase in LC3A/B expression has also been observed. The similar potentiation of autophagy in indole phytoalexin-treated cells has been reported recently. The study by Yang et al., 2023 suggested that autophagy was stimulated by enhancing LC3 expression in leukemic cells exposed to brassinin at a concentration of 50 μM [56]. In another study, bis-indole derivatives of 1-methoxyspirobrassinol methyl ether induced autophagy in A549 (lung carcinoma epithelial cells) by modulation of the phosphorylation or expression of various autophagy markers, such as Beclin-1, AMPK, ULK1, p62, Atg7, and LC3A/B [24]. The most notable observation from our experiments is the concentration-dependent increase in LC3A/B expression induced by MB-591 in cisplatin-resistant cells, indicating the substance’s ability to selectively induce autophagy in resistant ovarian cancer cells. In contrast, A2780 cells do not show significant changes in Beclin-1 expression, and ATG7 levels remain unchanged in both cell lines. This selective induction of autophagy in cisplatin-resistant cells opens up intriguing possibilities for targeted therapeutic interventions, especially in cases where resistance mechanisms present significant challenges.

To support or exclude the hypothesis regarding the role of ROS in the mechanism of MB action, we used NAC. N-acetylcysteine, a precursor of glutathione, is a widely used thiol-containing antioxidant and modulator of the intracellular redox state. The results in our study elucidate the interplay between MB-591 and NAC in modulating various cellular processes in both A2780 and A2780cis cells. NAC exhibits a protective effect against the cytotoxicity induced by MB-591, significantly affecting the viability of cells. When combined with MB-591, NAC counteracts alterations in cell cycle distribution, mitigating PCNA expression and decreasing p21 levels. Our analyses suggest an antiapoptotic effect of NAC, as it alleviated the generation of reactive oxygen species (ROS) induced by MB-591 in both ovarian cancer cell lines. Similar results were obtained by Hong et al. (2021), where NAC inhibited brassinin-induced ROS production and restored cellular proliferation in Huh7 (human hepatocellular carcinoma cells with mutated p53) and Hep3B (human hepatocellular carcinoma cells with deleted P53) [12]. In our experiments, NAC also prevents the MB-591-induced cleavage of PARP and reduces histone H2A.X phosphorylation, thereby revealing its protective role against DNA damage. Interestingly, NAC induces XIAP expression exclusively in A2780 cells. Cleaved caspase 9 levels decrease in A2780 cells, with a less pronounced effect in A2780cis cells, suggesting variable responses linked to cisplatin resistance cells. This paradoxical effect of NAC on apoptosis may depend on various factors, including the type of cells [67].

NAC demonstrates inhibitory effects on the PTEN/Akt/mTOR signaling pathway, particularly increasing the phosphorylation of PTEN in A2780cis cells. This antioxidant also interfered with the reduction in ULK1 phosphorylation induced by MB-591, indicating inhibition of autophagy initiation. Additionally, NAC significantly reduced the increase in LC3A/B protein expression induced by MB-591 in A2780cis cells, further highlighting its inhibitory role in autophagy. However, NAC did not prevent the decrease in Beclin-1 levels caused by MB-591, demonstrating a selective effect of MB-591 on autophagy in cisplatin-resistant cells.

As far as our understanding extends, this is the first report on the antiproliferative effects of 1-methoxyisobrassinin (MB-591) on sensitive and resistant ovarian cancer cells via ROS production and apoptosis induction. However, the current evidence is restricted to laboratory experiments, highlighting the need for further in vivo studies to comprehensively understand the potential of MB-591 as a chemotherapy option for ovarian cancer.

## 4. Materials and Methods

### 4.1. Tested Compound

The tested compound, 1-methoxyisobrassinin (MB-591) (Figure 1), was synthesized by Mariana Budovská at the Faculty of Science, P.J. Šafárik University, Košice. The synthesis of this compound was previously described in a study by Budovská et al., 2022 [26]. The compound was dissolved in DMSO. The final concentration of DMSO in the culture medium was kept below 0.2%, and it showed no cytotoxicity.

### 4.2. Cell Culture

The human cancer cell lines A2780 (human ovarian adenocarcinoma), A2780cis (human ovarian adenocarcinoma cisplatin-resistant cell line), and BJ-5ta (human dermal fibroblasts) were obtained from ATCC (Manassas, VA, USA). A2780 and A2780cis cell lines were cultured in RPMI 1640 growth medium (Biosera, Kansas City, MO, USA) supplemented with 10% fetal bovine serum (FBS) (Invitrogen, Carlsbad, CA, USA), and 1× HyClone™ Antibiotic/Antimycotic Solution (GE Healthcare, Piscataway, NJ, USA). BJ-5ta cells were cultured in a DMEM-M199 4:1 medium mixture, supplemented with 10% FBS and hygromycin B (0.01 mg/mL). Cells were maintained under standard conditions with an atmosphere containing 5% CO_2_ at 37 °C. Cell viability, estimated by trypan blue exclusion, was consistently greater than 95% before each experiment.

### 4.3. MTT Viability Assay

To determine the half-maximal inhibitory concentration (IC50) and confirm the antiproliferative effect of the tested compounds (MB-591, NAC, and cisplatin), we employed the MTT (3-(4,5-di-methylthiazol-2-yl)2,5-diphenyltetrazolium bromide) colorimetric assay from Sigma-Aldrich Chemie, Steinheim, Germany. The tested cell lines were seeded in 96-well culture plates at the following densities: A2780 (2 × 10^3^ cells/well), A2780cis (2.5 × 103 cells/well), and BJ-5ta (7.5 × 10^3^ cells/well). After 24 h, the tested compounds were added at the following concentrations: MB-591 (1, 3, 5, 7, 10, 20, 30, 50 and 100 μmol/L), NAC (0.1, 0.2, 0.5, 1, 2, and 2.5 mmol/L), and cisplatin (1, 2, 5, 10, 20, 30 and 50 μmol/L). The compounds were further incubated for 72 h after being added. Subsequently, 10 μL of MTT (5 mg/mL) was added to each well containing cells and incubated for an additional 4 h at 37 °C, during which MTT was metabolized to insoluble formazan within the cells. After 4 h, 100 μL of a 10% sodium dodecyl sulfate (SDS) solution was added to each well, and an additional 24 h were allowed for the formazan crystals to dissolve. The metabolic activity of the cells was evaluated by measuring absorbance at a wavelength of 540 nm using the automated Cytation™ 3 Cell Imaging Multi-Mode Reader (Biotek, Winooski, VT, USA). A nonlinear regression method was used to calculate IC50 values. Three independent analyses were performed.

### 4.4. 5-Bromo-2’-Deoxyuridine (BrdU) Cell Proliferation Assay

Cell proliferation activity was directly monitored by quantifying BrdU incorporated into the genomic DNA during cell growth. DNA synthesis was assessed using a colorimetric cell proliferation ELISA assay (Roche Diagnostics GmbH, Mannheim, Germany), following the vendor’s protocol. Briefly, the tested cell lines, at the same density as in the MTT viability assay, were plated in 96-well polystyrene microplates (Sarstedt AG & Co, Nümbrecht, Germany) with 80 µL of medium. Twenty-four hours after cell seeding, different concentrations (1, 3, 5, 7 and 10 μmol/L) of the compound MB-591 were added. After 48 h of treatment, cells were incubated with BrdU labeling solution (10 µM final concentration) for an additional 24 h at 37 °C, followed by fixation and incubation with anti-BrdU peroxidase conjugate for an additional 1.5 h at room temperature (RT). Finally, after the TMB substrate reaction, the stop solution (25 µL 1 mol/L H_2_SO_4_) was added. The change in absorbance was analyzed at 450 nm (for yellow) and 690 nm (for blue) using the automated CytationTM 3 Cell Imaging Multi-Mode Reader (Biotek, Winooski, VT, USA). Three independent experiments were performed.

### 4.5. Flow Cytometric Analyses

The cells were seeded at a density of 1.8 × 106 for A2780 and 3 × 106 for A2780cis in Petri dishes (6 cm) with complete growth medium and cultivated for 24 h. Following cell cultivation, the cancer cells were treated with MB-591 at concentrations of IC12.5, IC25, and IC50 μmol/L, with NAC (2.5 mmol/L) alone and in combination with MB-591 (IC50) for 3, 6, 12, 24, 48, and 72 h. Both cell lines were subjected to treatment with DMSO as the negative control at the same concentrations and for the same durations.

#### 4.5.1. Annexin V/PI Staining

Apoptosis is characterized by the externalization of phosphatidylserine, a process assessed through Annexin V/PI staining. The cell pellet was resuspended in 100 µL of PBS and incubated with Annexin V–Alexa Fluor^®^ 647 solution (1:300, Thermo Scientific, Rockford, IL, USA) for 15 min in the dark. After washing in PBS, 1 µL of PI (0.025 mg/mL) (Sigma Aldrich, St. Louis, MO, USA) was added to the samples. Data analysis was performed using the FL-2 (585/42) vs. FL-4 (661/16) channels, with a minimum of 1 × 104 events analyzed per sample. All experiments were conducted in triplicate, and FlowJo software v.10 (BD Biosciences, San Jose, CA, USA) was used for the analysis of FC data.

#### 4.5.2. Detection of Mitochondrial Membrane Potential Changes

Mitochondrial membrane potential (MMP) was assessed through the retention of the red-orange positively charged dye tetramethylrhodamine ethyl ester (final concentration 0.1 µmol/L TMRE; Molecular Probes, Eugene, OR, USA). Cells were treated with TMRE (Ex/Em 549/574 nm) and incubated for 30 min at RT, protected from light. The fluorescence signal was collected using the FL-2 (585/42) channel. Subsequent data analysis was conducted using FlowJo software v.10 (BD Biosciences, San Jose, CA, USA).

#### 4.5.3. Analysis of Cytochrome c Release

To investigate the release of cytochrome c, flow cytometric analysis was conducted employing the Cytochrome c Antibody (6H2) FITC Conjugate. The cellular population was fixed with 4% paraformaldehyde for 15 min, underwent PBS washing, and then underwent permeabilization with ice-cold 90% methanol. After a 15 min incubation on ice, the cells were washed in PBS and treated with the conjugated antibody for 30 min at RT in the dark. Following this, the cells were washed in PBS before analysis, and the fluorescence signal was acquired through the FL-1 (530/30) channel. The subsequent data analysis was carried out using FlowJo software v.10 (BD Biosciences, San Jose, CA, USA).

#### 4.5.4. Cell Cycle Analysis

For flow cytometric assessment of the cell cycle, both floating and adherent A2780 and A2780cis cells were collected at 72 h following treatment with the tested compounds. Subsequently, they were washed in PBS, fixed in cold 70% ethanol, and stored at −20 °C for more than 24 h. Before analysis, the cells underwent two PBS washes, were resuspended in a staining solution (0.1% Triton X-100 in PBS, ribonuclease A (0.137 mg/mL), and propidium iodide (0.02 mg/mL), and were incubated in the dark for 30 min at RT. Analysis was then carried out using a flow cytometer (BD FACSCalibur, BD Biosciences, San Jose, CA, USA). The FL-2 (585/42) channel was utilized for collecting the fluorescence signal. A minimum of 1 × 104 events were analyzed per sample, and all experiments were conducted in triplicate. Cell aggregates were identified using FL-2-W vs. FL-2-A plots. Histograms and cell population markers were generated using FlowJo software v.10 (BD Biosciences, San Jose, CA, USA), applying the Dean–Jett–Fox model for all samples.

#### 4.5.5. Flow Cytometry Analysis of ROS

A2780 and A2780cis cell lines were seeded in Petri dishes and incubated for 24 h in a complete medium containing 10% fetal bovine serum (FBS). Subsequently, the cells were exposed to IC50 of MB-591, as well as to NAC (2.5 mmol/L) alone and in combination with MB-591. These treatments were administered at time intervals of 3, 6, 12, 24, 48, and 72 h prior to subsequent analysis. The antioxidant NAC was employed as a pre-treatment for 30 min before the addition of MB-591. Following treatment, both floating and adherent cells were collected, washed in phosphate-buffered saline (PBS), allocated for specific analyses, and subjected to staining with DHR123 (dihydrorhodamine 123) at final concentration 200 nM. After a 15 min incubation at RT in the absence of light, the samples were placed on ice, and fluorescence alterations were assessed using a BD FACSCalibur flow cytometer (BD Biosciences). A minimum of 1 × 104 events were analyzed per assessment.

### 4.6. Western Blot Analyses

A2780 and A2780cis ovarian cancer cells underwent exposure to varying concentrations (IC 12.5, IC 25, and IC 50 μmol/L) of MB-591, as well as to NAC (2.5 mmol/L) either alone or in combination with MB-591 for a duration of 72 h. Protein lysates from the treated cells were prepared utilizing a Laemmli lysis buffer, consisting of glycerol, 1M Tris/HCl (pH 6.8), 20% sodium dodecyl sulfate (SDS), deionized H20, phosphatase and protease inhibitors (Sigma-Aldrich), employing a sonication process. The concentration of proteins was determined using the Pierce^®^ BCA Protein Assay Kit (Thermo Scientific, Rockford, IL, USA) and quantified by an automated Cytation™ 3 Cell Imaging Multi-Mode Reader (Biotek) at a wavelength of 570 nm. Subsequently, proteins (25–40 μg of sample per well) were electrophoresed on a SDS-PAA gel (10%) at 100 V for 2.5 h and then transferred to a polyvinylidene difluoride (PVDF) membrane using the iBlot™ 2 Dry Blotting System (Invitrogen, Carlsbad, CA, USA). The membranes, with transferred proteins, underwent blocking in 5% BSA (bovine serum albumin; SERVA, Heidelberg, Germany) or 5% non-fat dry milk (Cell Signaling Technology^®^, Danvers, MA, USA) in TBS-Tween (pH 7.4) for 1 h at RT to minimize non-specific binding. Following blocking, incubation with primary antibodies (refer to Table 4) occurred overnight at 4 °C. On the subsequent day, membranes were washed in TBS-Tween (3 × 5 min) and incubated with the corresponding horseradish peroxidase (HRP)-conjugated anti-mouse or anti-rabbit secondary antibody for 1 h at RT. Following incubation, membranes were washed again in TBS-Tween (3 × 5 min), and the expression of proteins was detected using the iBright TM FL1500 Imaging System with chemiluminescent ECL substrate (Thermo Scientific, Rockford, IL, USA). A densitometric analysis of Western blot (WB) results was conducted using the Image Studio™ Lite Software (LI-COR Biosciences, Lincoln, NE, USA). Equal loading was verified using the β-actin antibody. The entire process was independently performed three times.

### 4.7. Statistical Analysis

The results of our experiments are presented in the form of mean values accompanied by the standard error of the mean (SEM), derived from a minimum of three distinct and independent experimental trials. Statistical analysis to assess the significance between different groups was carried out utilizing a one-way analysis of variance (ANOVA), incorporating Tukey’s multiple comparison test. Throughout this document, * indicates *p* < 0.05, ** *p* < 0.01, *** *p* < 0.001 vs. DMSO treated negative control; # *p* < 0.05, ## *p* < 0.01, ### *p* < 0.001 vs. MB-591 treated cells. 

## 5. Conclusions

The present study demonstrated the significant antiproliferative properties of 1-methoxyisobrassinin (MB-591), a regioisomer of the natural indole phytoalexin 1-methoxybrassinin, exhibited in both sensitive and cisplatin-resistant ovarian cancer cells in vitro. In both cell lines, MB-591 induced ROS production associated with the induction of apoptotic cell death machinery. ROS-induced DNA damage led to alterations in the distribution of the cell cycle, particularly in the S and G2/M phases. These changes were accompanied by modifications in key cell cycle regulatory proteins, activation of caspase-9, PARP cleavage, and mitochondrial dysfunction. The role of ROS in the antiproliferative effect of MB-591 was confirmed by a set of experiments with NAC. This strong antioxidant significantly suppressed not only ROS production but also several processes leading to apoptosis.

Furthermore, modulation of expression/phosphorylation of proteins such as LC3A/B, ULK1 or PTEN in resistant A2780cis cells indicates possible activation of autophagy. The induction of autophagy, however, ultimately failed to prevent cancer cell apoptosis. We believe that the activation of autophagy may represent an attempt by resistant tumor cells to survive. Nevertheless, this hypothesis requires further exploration and validation through additional investigation.

## Figures and Tables

**Table 1:** IC50 (µmol/L) of MB-591 and cisplatin of A2780, A2780cis and Bj-5ta cell lines after 72 h of incubation.

Compound	Assay		Cell Line	
		A2780	A2780cis	Bj-5ta
**MB-591**	MTT	3.62 ± 1.19	7.00 ± 0.82	27.43 ± 2.42
	BrdU	5.41 ± 1.27	7.06 ± 1.09	18.11 ± 0.93
	Selectivity index	7.57/3.34	3.91/2.56	
**CisPt**	MTT	1.64 ± 0.35	12.73 ± 2.55	31.3 ± 3.86
	Selectivity index	19.08	2.45	

**Table 2:** Cell cycle analysis of A2780 (**A**) and A2780cis (**B**) ovarian cancer cells after treatment with MB-591 for 72 h at IC12.5, IC25 and IC50 concentration.

**A**	**G1**	**S**	**G2/M**
**DMSO**	61.7 ± 2.8	17.3 ± 2.6	22.4 ± 2.4
**IC12.5**	59.7 ± 2.4	19.1 ± 2.4	22.5 ± 3.6
**IC25**	61.9 ± 0.4	25.1 ± 0.2 *	13.45 ± 0.7 *
**IC50**	56.1 ± 3.5	17.7 ± 2.9	26.3 ± 2.7
**B**	**G1**	**S**	**G2/M**
**DMSO**	61.03 ± 2.57	22.53 ± 2.41	16.53 ± 2.67
**IC12.5**	62.95 ± 4.17	11.08 ± 4.12 *	26.66 ± 4.44 *
**IC25**	64.65 ± 0.21	25.3 ± 5.3	10.20 ± 4.8
**IC50**	41.4 ± 3.55 **	29.5 ± 2.82 *	28.2 ± 3.11 **

Data show mean plus minus SD of three independent experiments. Statistical significance: * *p* < 0.05, ** p < 0.01, vs. DMSO (vehicle).

**Table 3:** Cell cycle analysis of A2780 (**A**) and A2780cis (**B**) cells after 3, 6, 12, 24, 48 and 72 h of incubation with MB-591, NAC and NAC/MB-591.

**A**		**G1**	**S**	**G2/M**
3 h	**DMSO**	47.1 ± 0.1	31.7 ± 1.8	21.95 ± 3.6
	**MB-591**	50.65 ± 1.6	30.5 ± 4.2	19.25 ± 1.1
	**NAC**	47.9 ± 1.3	30.2 ± 5.8	21.1 ± 5.7
	**NAC/MB-591**	53.85 ± 0.1 ***	30.05 ± 4.3	15.1 ± 4.4
6 h	**DMSO**	52.05 ± 0.07	27.95 ± 0.63	19.05 ± 0.7
	**MB-591**	44.05 ± 4.2	40.05 ± 2.0 **	15.1 ± 4.0
	**NAC**	57.65 ± 1.48 *	24.4 ± 6.5	18.1 ± 3.95
	**NAC/MB-591**	54.95 ± 1.34	24.25 ± 3.6 ##	19.9 ± 2.4
12 h	**DMSO**	53.55 ± 0.35	23.6 ± 0.14	18.1 ± 0.56
	**MB-591**	53.75 ± 1.9	6.20 ± 2.22 **	39.9 ± 0.42 ***
	**NAC**	56.15 ± 1.48	24.25 ± 1.9	20.95 ± 2.61
	**NAC/MB-591**	55.55 ± 0.21 *	23.4 ± 3.95 #	19.5 ± 3.99 #
24 h	**DMSO**	49.1 ± 3.4	33.6 ± 2.8	17.3 ± 2.6
	**MB-591**	42.3 ± 4.2	22.3 ± 2.8 *	35.4 ± 4.6 **
	**NAC**	52.1 ± 2.1	26.0 ± 2.0	21.1 ± 2.7
	**NAC/MB-591**	52.45 ± 0.1	26.2 ± 3.4	21.15 ± 3.5
48 h	**DMSO**	58.86 ± 3.61	24.03 ± 5.7	16.76 ± 4.24
	**MB-591**	39.67 ± 3.05 **	24.8 ± 2.8	35.30 ± 1.83 *
	**NAC**	56.26 ± 4.9	25.6 ± 5.7	18.58 ± 1.11
	**NAC/MB-591**	57.7 ± 0.45 ###	19.7 ± 3.9	22.67 ± 4.07 #
72 h	**DMSO**	61.7 ± 2.81	17.36 ± 2.65	22.4 ± 2.98
	**MB-591**	56.1 ± 3.5	17.7 ± 2.9	26.3 ± 2.7
	**NAC**	60.0 ± 0.84	19.1 ± 2.55	21.87 ± 2.7
	**NAC/MB-591**	62.1 ± 4.4	18.96 ± 4.3	18.26 ± 3.0
**B**		**G1**	**S**	**G2/M**
3 h	**DMSO**	51.2 ± 2.26	26.3 ± 3.25	21.65 ± 0.91
	**MB-591**	39.76 ± 5.19	50.15 ± 4.03 **	9.31 ± 3.8 **
	**NAC**	46.45 ± 0.21	29.35 ± 2.19	23.4 ± 0.42
	**NAC/MB-591**	52.8 ± 0.34	24.86 ± 1.5	21.1 ± 1.9
6 h	**DMSO**	52.25 ± 0.07	22.3 ± 3.11	24.3 ± 3.81
	**MB-591**	46.35 ± 2.33	18.5 ± 3.52	34.9 ± 1.69
	**NAC**	52.1 ± 1.5	25.1 ± 3.2	21.36 ± 2.03
	**NAC/MB-591**	53 ± 1.41	24.15 ± 5.44	23.0 ± 5.6
12 h	**DMSO**	54.93 ± 1.2	27.66 ± 3.06	16.76 ± 1.88
	**MB-591**	49.2 ± 1.69 *	23.1 ± 1.27	28.6 ± 2.26 **
	**NAC**	60.46 ± 1.96 *	16.06 ± 5.75	23.06 ± 4.65
	**NAC/MB-591**	55.2 ± 1.55	29.4 ± 3.65	16.25 ± 1.76 #
24 h	**DMSO**	51.63 ± 1.92	28.23 ± 2.95	19.07 ± 1.62
	**MB-591**	35.33 ± 4.85 **	36.06 ± 4.5	29.36 ± 3.17 ##
	**NAC**	50.36 ± 3.26	28.0 ± 2.42	21.03 ± 1.7
	**NAC/MB-591**	46.96 ± 2.9 #	30.46 ± 5.7	22.8 ± 1.11 ##
48 h	**DMSO**	56.36 ± 0.49	23.86 ± 0.28	19.83 ± 0.85
	**MB-591**	29.95 ± 0.21 ***	29.86 ± 2.66 *	38.3 ± 1.8 ***
	**NAC**	51.63 ± 1.83 *	24.16 ± 1.04	22.46 ± 3.59
	**NAC/MB-591**	56.8 ± 1.21 ###	19.76 ± 1.9 *	24.46 ± 3.35 ##
72 h	**DMSO**	61.03 ± 2.57	22.53 ± 2.41	16.53 ± 2.67
	**MB-591**	41.4 ± 3.55 **	29.5 ± 2.82 *	28.2 ± 3.11 **
	**NAC**	57.56 ± 0.45	23.4 ± 0.52	17.8 ± 0.51
	**NAC/MB-591**	41.4 ± 3.66 **	29.5 ± 2.82 *	26.2 ± 3.92 *

Results are presented from three independent measurements as mean ± standard deviation. Statistical significance: * *p* < 0.05, ** *p* < 0.01, *** *p* < 0.001 vs. negative control; *# p* < 0.05, ## *p* < 0.01, ### *p* < 0.001 vs. MB-591.

**Table 4:** List of Western blot antibodies.

**Primary Antibody**	**MW (kDa)**	**Origin**	**Dilution**	**Catalogue No.**	**Manufacturer**
PCNA (D3H8P) XP^®^ Rabbit mAb	36	Rabbit	1:1000	#13110	Cell Signaling Technology, Danvers, MA, USA
p21 Waf1/Cip1 (12D1) Rabbit mAb	21	Rabbit	1:1000	#2947	
Phospho-Cyclin B1 (Ser133) (9E3) Rabbit mAb	55	Rabbit	1:1000	#4133	
Cyclin B1 (D5C10) Rabbit mAb	55	Rabbit	1:1000	#12231	
Phospho-Rb (Ser807/811) (D20B12) XP Rabbit mAb	110	Rabbit	1:1000	#8516	
PARP (46D11) Rabbit mAb	116/89	Rabbit	1:1000	#9091	
Phospho-Histone H2A.X (Ser139) (20E3) Rabbit mAb	15	Rabbit	1:1000	#9718	
XIAP (3B6) Rabbit mAb	53	Rabbit	1:1000	#2045	
Cleaved Caspase-9 (Asp315) (D8I9E) Rabbit mAb	35	Rabbit	1:1000	#20750	
Phospho-PTEN (Ser380) Antibody	54	Rabbit	1:1000	#9551	
Phospho-Akt (Thr308) (244F9) Rabbit mAb	60	Rabbit	1:1000	#4056	
Phospho-mTOR (Ser2448) (D9C2) XP Rabbit mAb	289	Rabbit	1:1000	#5536	
mTOR (7C10) Rabbit mAb	289	Rabbit	1:1000	#2983	
Phospho-ULK1 (Ser757) (D7O6U) Rabbit mAb	140–151	Rabbit	1:1000	#14202	
Beclin-1 (D40C5)	60	Rabbit	1:1000	#3495	
Atg7 (D12B11) Rabbit mAb	78	Rabbit	1:1000	#8558	
LC3A/B (D3U4C) XP^®^ Rabbit mAb	15–18	Rabbit	1:1000	#12741	
β-Actin (8H10D10) Mouse mAb	45	Mouse	1:1000	#3700	
APAF1 Recombinant Rabbit Monoclonal Antibody (SY22-02)	135	Rabbit	1:1000	MA5-32082	Thermo Scientific
**Secondary Antibody**	**MW (kDa)**	**Origin**	**Dilution**	**Catalogue No.**	**Manufacturer**
Anti-rabbit IgG, HRP-linkedAntibody	-	Goat	1:1000	#7074	Cell Signaling Technology, Danvers, MA, USA
Anti-mouse IgG, HRP-linked Antibody	-	Goat	1:1000	#7076	

## Data Availability

Data are contained within the article and Supplementary Materials.

## Supplementary Materials

The following supporting information can be downloaded at: https://www.mdpi.com/article/10.3390/molecules29081773/s1, Figure S1: Representative histograms of cell cycle distribution in A2780 and A2780cis cells treated with MB-591 at concentrations of IC12.5, IC25 and IC50 after 72 h; Figure S2: Representative dot-blot diagrams of changes in MMP in A2780 and A2780cis cells after 72 h treatment with with MB-591 at IC12.5, IC25 and IC50 concentrations; Figure S3: Representative dot-blot diagrams of Annexin V/PI staining in A2780 and A2780cis cells after 72 h treatment with with MB-591 at IC12.5, IC25 and IC50 concentrations; Figure S4: Representative histograms of cell cycle distribution in A2780 (A) and A2780cis (B) cells treated with MB-591, NAC and NAC/MB-591 after 3, 6, 12, 24, 48 and 72 h; Figure S5: Representative dot-blot diagrams of changes in MMP in A2780 (A) and A2780cis (B) cells treated with MB-591 at concentration of IC50, NAC and NAC/MB-591 after 3, 6, 12, 24, 48 and 72 h of incubation; Figure S6: Representative dot-blot diagrams of Annexin V/PI staining in A2780 (A) and A2780cis (B) cells after treatment with MB-591 at concentration of IC50, NAC and NAC/MB-591 after 3, 6, 12, 24, 48 and 72 h.
