# Supplementary material for: Exploring the Antiproliferative and Modulatory Effects of 1-Methoxyisobrassinin on Ovarian Cancer Cells: Insights into Cell Cycle Regulation, Apoptosis, Autophagy, and Its Interactions with NAC
Source: Molecules. 2024 Apr 13;29(8):1773. doi: 10.3390/molecules29081773 (PMC11052400; doi:10.3390/molecules29081773)
Supplement: Supplementary file 1 [file molecules-29-01773-s001.zip › molecules-2875984-supplementary.pdf]

Exploring the Antiproliferative and Modulatory Effects of 1-Methoxyisobrassinin on Ovarian Cancer Cells: Insights into Cell Cycle Regulation, Apoptosis, Autophagy, and Its Interactions with NAC

Martina Zigová [1], Viktória Miškufová [1], Marianna Budovská [2], Radka Michalková [1,*] and Ján Mojžiš [1,*]

[1] Department of Pharmacology, Faculty of Medicine, Pavol Jozef Šafárik University, 040 01 Košice, Slovakia; chripkova.martina@gmail.com (M.Z.); viktoria.miskufova@student.upjs.sk (V.M.)
[2] Department of Organic Chemistry, Institute of Chemistry, Faculty of Science, Pavol Jozef Šafárik University, 040 01 Košice, Slovakia; marianna.budovska@upjs.sk
* Correspondence: radka.michalkova@upjs.sk (R.M.); jan.mojzis@upjs.sk (J.M.)

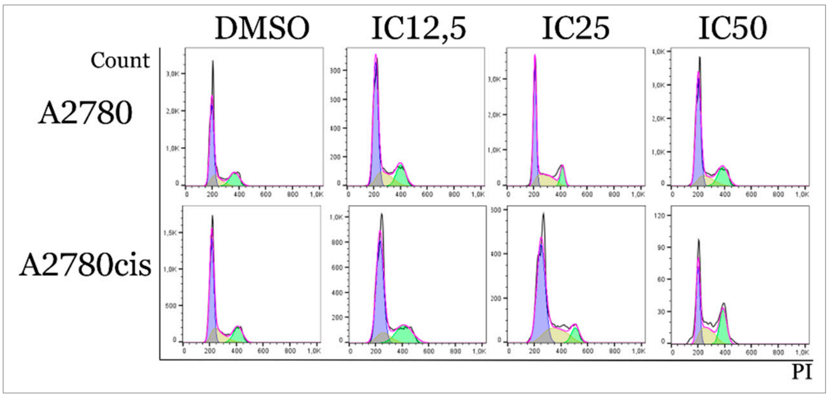

Figure S1. Representative histograms of cell cycle distribution in A2780 and A2780cis cells treated with MB-591 at concentrations of IC12.5, IC25 and IC50 after 72 h.

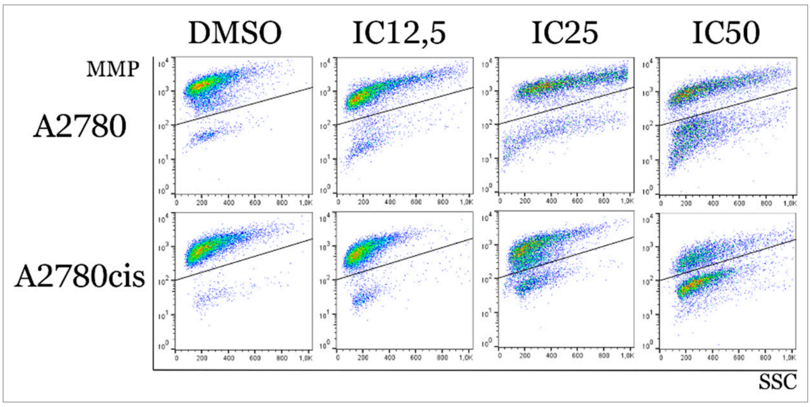

Figure S2. Representative dot-blot diagrams of changes in MMP in A2780 and A2780cis cells after 72h treatment with with MB-591 at IC12.5, IC25 and IC50 concentrations.

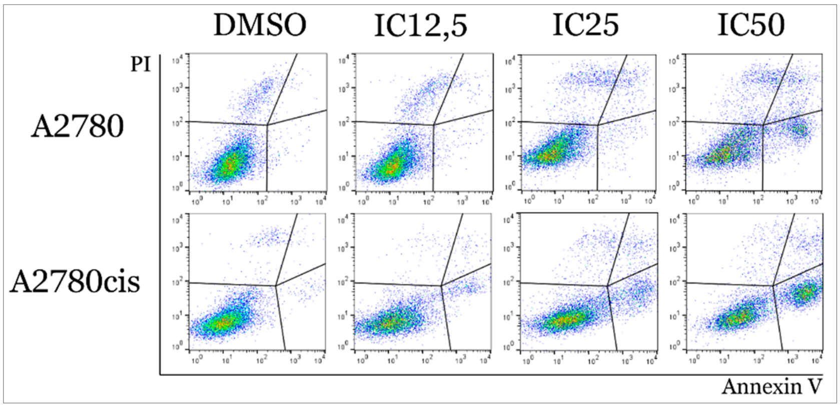

Figure S3. Representative dot-blot diagrams of Annexin V/PI staining in A2780 and A2780cis cells after 72h treatment with with MB-591 at IC12.5, IC25 and IC50 concentrations**.**

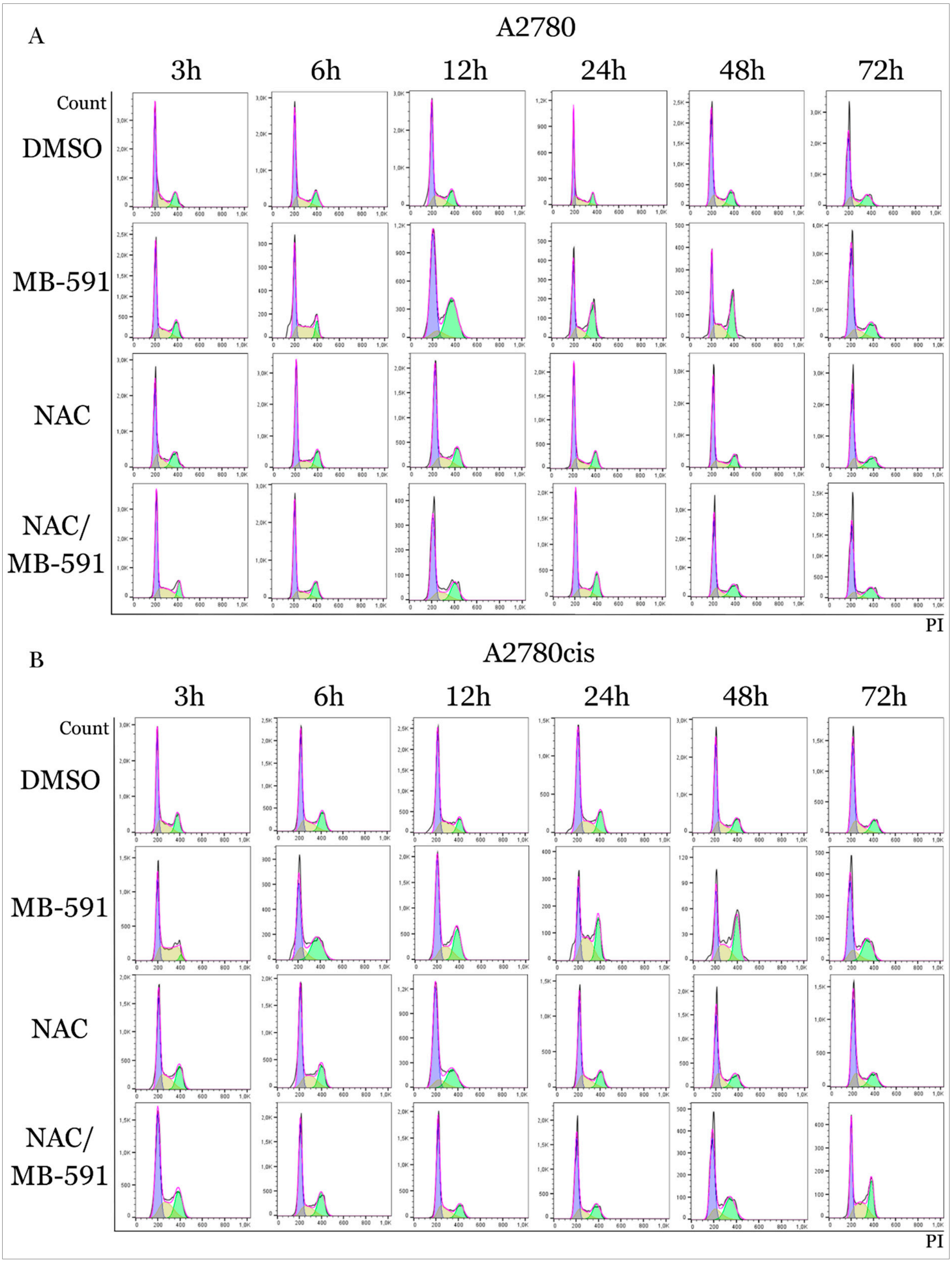

Figure S4. Representative histograms of cell cycle distribution in A2780 (**A**) and A2780cis (**B**) cells treated with MB-591, NAC and NAC/MB-591 after 3, 6, 12, 24, 48 and 72h.

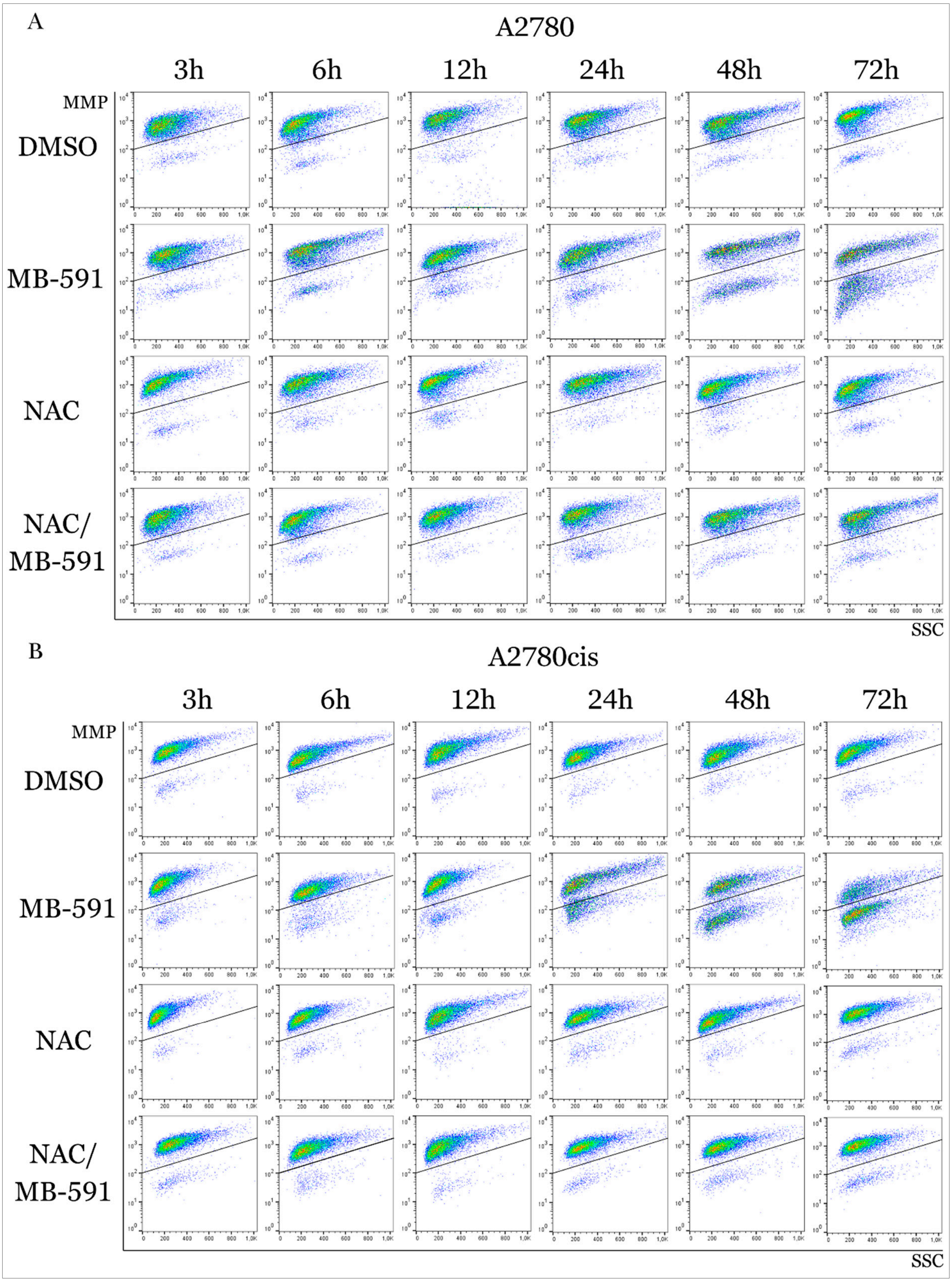

Figure S5. Representative dot-blot diagrams of changes in MMP in A2780 (**A**) and A2780cis (**B**) cells treated with MB-591 at concentration of IC50, NAC and NAC/MB-591 after 3, 6, 12, 24, 48 and 72h of incubation.

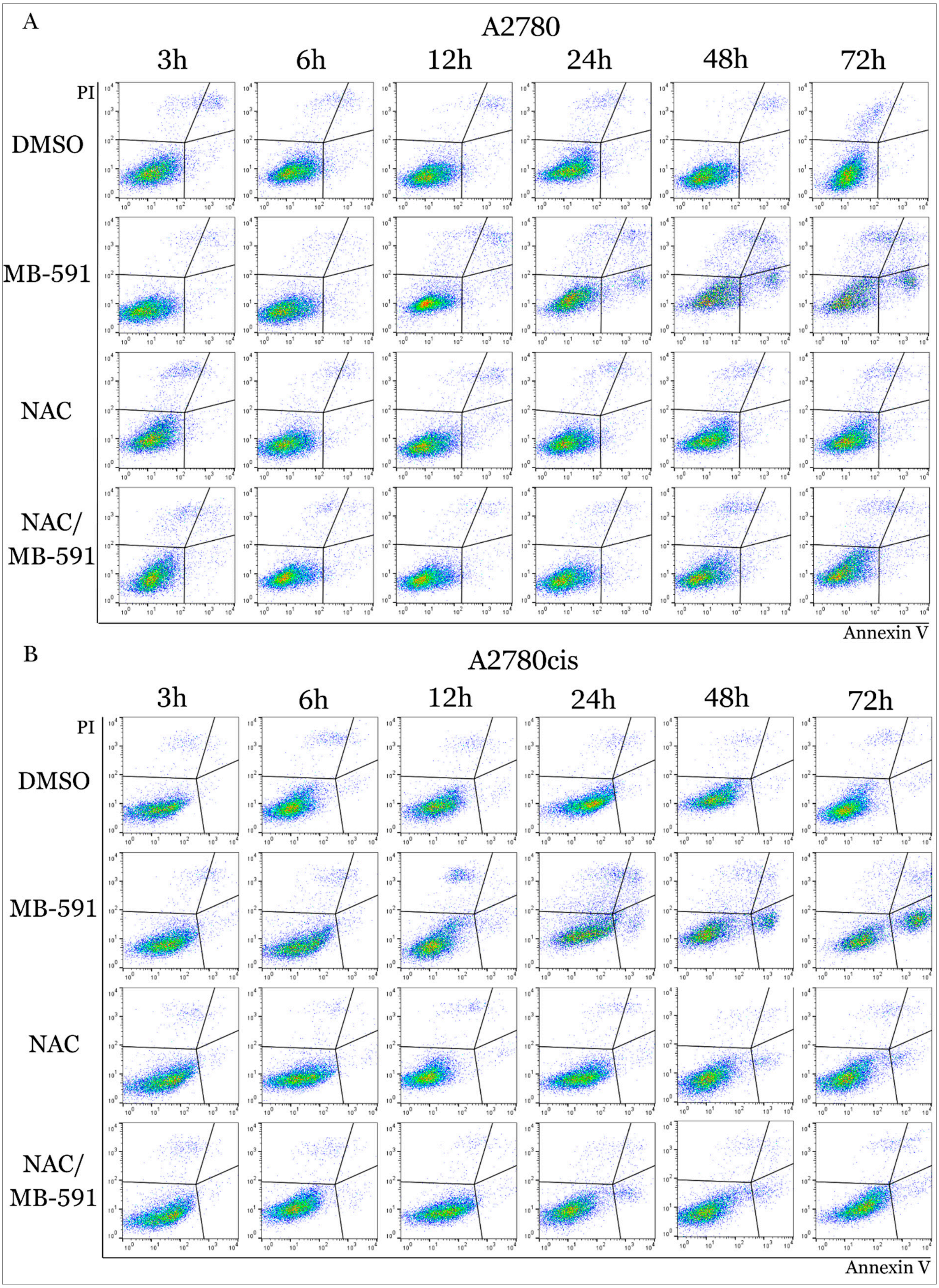

Figure S6. Representative dot-blot diagrams of Annexin V/PI staining in A2780 (**A**) and A2780cis (**B**) cells after treatment with MB-591 at concentration of IC50, NAC and NAC/MB-591 after 3, 6, 12, 24, 48 and 72h.
